# Kinetics and process optimization studies for the effective removal of cresyl fast violet dye using reusable nanosized mullite

**DOI:** 10.1038/s41598-024-81653-y

**Published:** 2024-12-31

**Authors:** Omar A. Fouad, Yara M. Adly, Wafaa M. Hosny, Gehad G. Mohamed, Maysa R. Mostafa

**Affiliations:** 1 Faculty of Science, Chemistry Department, Cairo University, Giza, 12613 Egypt; 2 Nanoscience Department, Institute of Basic and Applied Sciences, Egypt-Japan University of Science and Technology, New Borg El Arab, Alexandria, 21934 Egypt

**Keywords:** Cresyl fast violet (CFV), Adsorption, Nanoceramic mullite, Langmuir, Freundlich Isotherm, DKR, SEM, TEM, EDX, Zeta potential, Nanoparticles, Environmental monitoring, Pollution remediation, Nanoparticle synthesis

## Abstract

The swift rise of hazardous dye effluent from diverse sectors continues to be a severe public health problem and a top priority for environmental preservation, presenting a significant obstacle to the current conventional water treatment systems. This study aims to develop an efficient and reusable approach for removing cresyl fast violet dye using mullite nanoparticles. Some factors such as pH, nano-mullite dosage, agitation speed, time, and others that affect the removal process were studied. The mullite nanoparticles’ shape, particle size, pore diameters, and crystal phase structure are characterized using many techniques such as Brunauer-Emmett-Teller (BET) surface area, X-ray diffraction (XRD), transmission electron microscopy (TEM), Contact angle, Zeta potential, scanning electron microscope (SEM) as well as energy dispersive X-ray analysis (EDX). The optimal conditions were pH 7 and 600 rpm for 30 min at room temperature. Using statistical programs such as ANOVA and Design Expert, the dye removal parameters were modeled and optimized, where the removal percentage was about 99%. In addition, the experimental elimination process exceeded 90% after just 10 min. Langmuir, Freundlich, Dubinin-Kaganer-Raduskevich (DKR), and Temkin isotherm equations were examined to find the adsorption isotherm. The experimental data fits the pseudo-second-order model and the Freundlich isotherm. Thermodynamic investigations confirmed that the adsorption process was endothermic and spontaneous. The nano-mullite was employed for the removal process, and its recycling ability supports its economic benefits. It was found that the high percentage of elimination remained consistent for more than 3 cycles.

**Supplementary Information:**

The online version contains supplementary material available at 10.1038/s41598-024-81653-y.

## Introduction

**Fig. 1:**
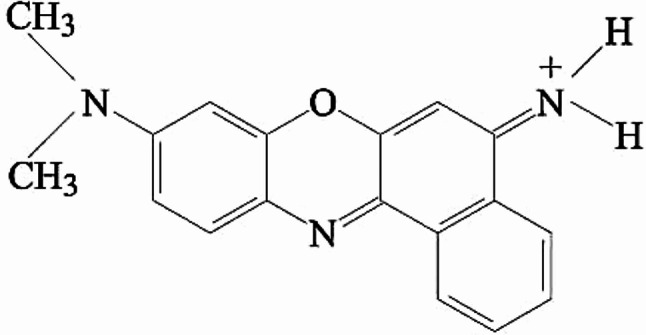
Structure of cresyl fast violet.

Many industries use synthetic dyes, including the food and pharmaceutical sectors and the textile processing industry. However, about 80% of the wastewater produced from these industries contains dyes that are released untreated into rivers or applied directly to farming^1^. Organic dyes are thought to be serious water pollutants. Dye exposure has been linked to severe health risks for humans and other organisms. It may cause liver, brain, nervous system, renal and reproductive dysfunction^2^. Given the rising demand for water and the contamination of the environment, this poses a threat not only to humans but to all living things^3^. Especially, the basic and diazo direct dyes showed the highest rates of toxicity. Wastewater treatment has shifted its focus to the printing and dyeing industries, which use dyes for coloring^4^. One of these dyes is (9-amino-5-imino-5 H-benzo[a] phenoxazine), which is called cresyl fast violet as in Fig. 1^5^. Also, it is considered a popular oxazine basic pigment which exhibit cationic properties when dissolved in water and react on their basic sides^6,7^. It has optical microscopic studies of tissues in medicine and biology in addition to it stains the nissl substance (rough endoplasmic reticulum), neurons, and glia because its adsorption ability as adsorbate as (Stereotaxic Brain Atlas of the Egyptian Fruit Bat 2021). The substance is a dark green powder with a crystalline structure. It may easily dissolve in water, making it very mobile in ecological environments. It poses risks when inhaled or ingested. The hazardous material’s data sheet indicates that the principal entry routes are through the eyes, ingestion, inhalation, and skin. It induces irritation in the eyes and skin. Signs of poisoning may manifest even after several hours, so it is imperative to undergo medical surveillance for a minimum of 48 h following the incident^8^. There aren’t many prior studies on the adsorption of the cresyl violet dye; nevertheless, there are studies on the adsorption of the dye on a nanomaterial made by oxidizing graphite oxide and then sonicating it with a capacity of roughly q = 190 mg/g in about 20 min^9^.

There are numerous methods for removing these chemicals from the environment; most are based on methods utilizing biology, chemistry, and physics^10,11^. Various methods, including membrane filtration, adsorption, precipitation, coagulation, microbial biodegradation^12^, and ion- exchange^13^, have been employed to treat wastewater. However, many technologies have downsides, such as high operating and maintenance costs, hazardous sludge formation, and complex treatment procedures^14^. Thus, it is critical to research fresh and effective treatment options. Still, the adsorption technique is the most efficient of them all. It is capable of purifying wastewater and recycling organic dyes. Furthermore, there are low prices, simple operations, no harmful ingredients, and excellent process efficiency^12,15,16^. Physical force or chemical binding can trap targeted pollutants, whereas the surface area, specific active site, and selectivity of a porous adsorbent influence water contaminant adsorption. For nanoparticles to be efficient in water treatment, their size range, surface qualities, porosity, surface area to volume ratio, and other features must all be carefully managed. The skill of preparation and exact control of process parameters are critical in achieving these ideal features. Ceramic nanoparticles are used in industry as catalysts, filters, and lightweight structural supports in addition to they have been effectively applied to water treatment. Ceramic nanomaterials, which are inorganic systems with porous features, are mostly composed of metal oxides, phosphates, carbides, and carbonates, such as silicon, calcium, and titanium. They have a wide range of applications because to their numerous advantages, including good heat resistance and chemical inertness^15^.

Ceramic materials also provide more active areas for removing pollutants from water. Ceramics, such as mullite its general formula for mullite, an alumina silicate, is Al_4+2x_Si_2−2x_O_10−x_, where 0 < x < 1^17^. Nevertheless, mullites are scientifically intriguing at temperatures lower than 1873 K, with equilibrium compositions ranging from approximately 0.25 (3/2-mullite, 3Al_2_O_3_·2SiO_2_) to approximately 0.4 (2/1-mullite). At a temperature equal to or greater than 1873 K, the compound 2Al_2_O_3_·SiO_2_ was identified^18^. Mullite exhibits outstanding characteristics such as excellent heat resistance, durability, resistance to oxidation, a low coefficient of thermal expansion, high-temperature strength, superior resistance to thermal shock, and reduction in high-temperature creep, and it functions effectively as reinforcement^19^. Mullite ceramics are used in various industries, including manufacturing crucibles, heat exchangers, silicon solar cell substrates, dental ceramic components, hot gas filters, and electronic packaging^20^.

The mullite synthesis process is used to identify the precise proportion of mixture for both silicon and aluminum in the precursor, directly impacting the temperature at which mullitization occurs^21^. Many methods were used for the preparation of mullite nanoparticles, such as gel-casting^22^, freeze-casting^23^, direct-foaming^24^, foam-casting^25^, starch consolidation^26^, replica^27^, thermal gelation^28^ and sol-gel method^29^. These ceramic nanoparticles were made using the sol-gel technique^27^. This method enables thorough amalgamation to enhance the uniformity of the fundamental constituents, resulting in monophasic gels with an exact atomic-level elemental distribution. Statistical design software, such as ANOVA and Design Expert, utilizes experimental data to solve equations involving several variables. It evaluates data gathered at different locations in the design space and optimizes all process parameters to help forecast, enhance, and perfect a system’s behavior. It overcomes the constraints of traditional experimental techniques and examines the combined impact of all variables.

This study demonstrated the synthesis of the nano mullite by sol-gel method, followed by calcination at 1000 °C, then characterized by XRD, SEM, EDX, TEM, Contact angle, Zeta potential and BET studies. The prepared nano mullite was used as an adsorbent to remove cresyl fast violet dye depending on optimization of the factors affecting the removal using programs such as ANOVA and design expert to optimize and characterize the factors affecting the adsorption process of CFV dye on nano mullite with time and material saving. Determining the adsorption kinetics, isotherms reusability, and thermodynamics are among the goals.

## Discussions and results

### XRD analysis

**Fig. 2:**
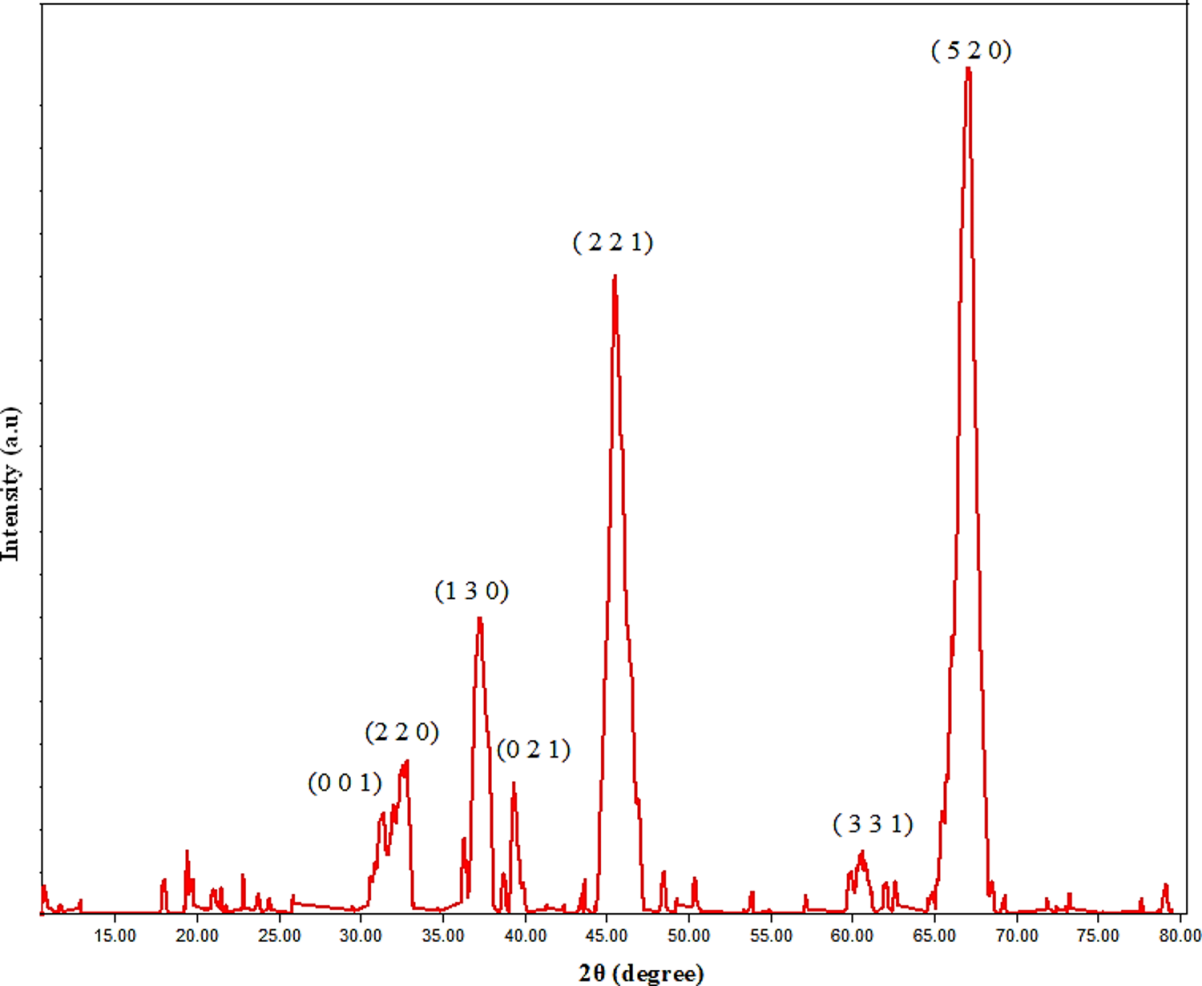
The X-ray diffraction patterns of the nano mullite.

Nano-mullite (3Al_2_O_3_.2SiO_2_), which was synthesized by sol-gel method followed by firing silicon and aluminum oxides, was confirmed by XRD as shown in Fig. (2); its XRD pattern revealed its structure, which is characterized by orthorhombic crystals in the P b a m (55) space group with card number 7105575^29^. The 2θ angles of the distinct diffraction peaks at 31.06°, 33.07°, 36.84°, 39.04°, 46.23°, 60.24°, and 66.20° are illustrated in Fig. 2. These peaks correspond to the (0 0 1), (2 2 0), (1 3 0), (0 2 1), (2 2 1), (3 3 1), and (5 2 0) planes. The nano-sized structure of the mullite nanoparticles was established utilizing the Debye-Scherrer equation. The average size of the crystallites ranged from 7 to 12 nm, with an average of 9.8 nm^29–32^.

### SEM and EDX of nano mullite

**Fig. 3:**
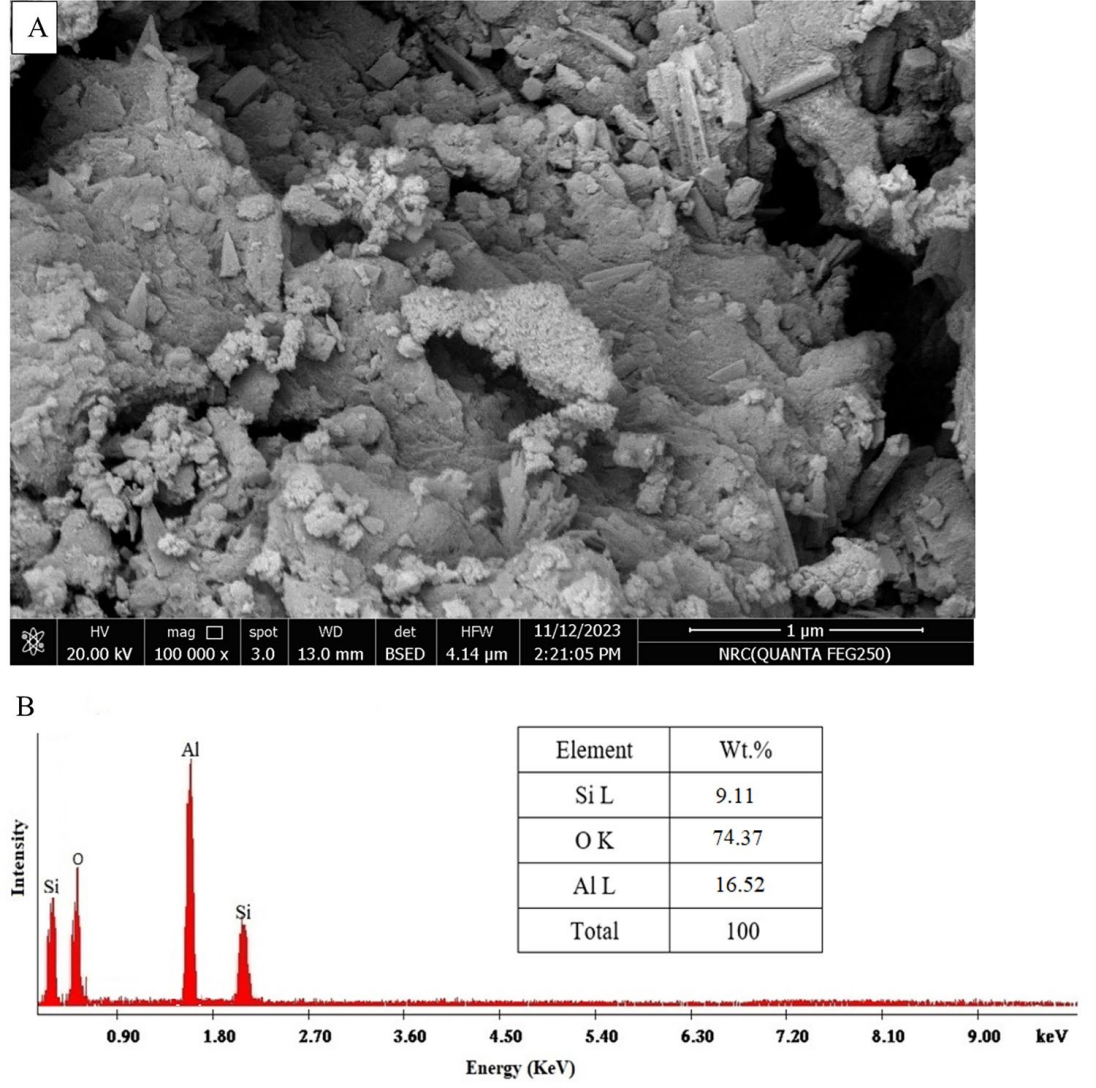
The SEM image (**A**) and EDX data (**B**) of the prepared mullite nanoparticles.

SEM pictures (Fig. 3A) showed a smooth surface, porous structure, consistent matrix, and irregularly shaped mullite nanoparticles. The synthesized mullite nanoparticles demonstrated the presence of various particle and pore sizes relying on SEM microphotographs. Energy dispersive X-ray analysis (EDX) is performed in conjunction with scanning electron microscopy (SEM) to provide detailed information about the elemental composition of a sample’s surface. EDX analyses have verified the accuracy of the chemical composition of nano mullite by revealing the composition (oxygen, aluminum, and silica) and their weight percentages (Fig. 3B). The composition comprises approximately 9.11% silica, 16.52% aluminum, and 74.37% oxygen.

### Transmission electron microscopy (TEM) study

**Fig. 4:**
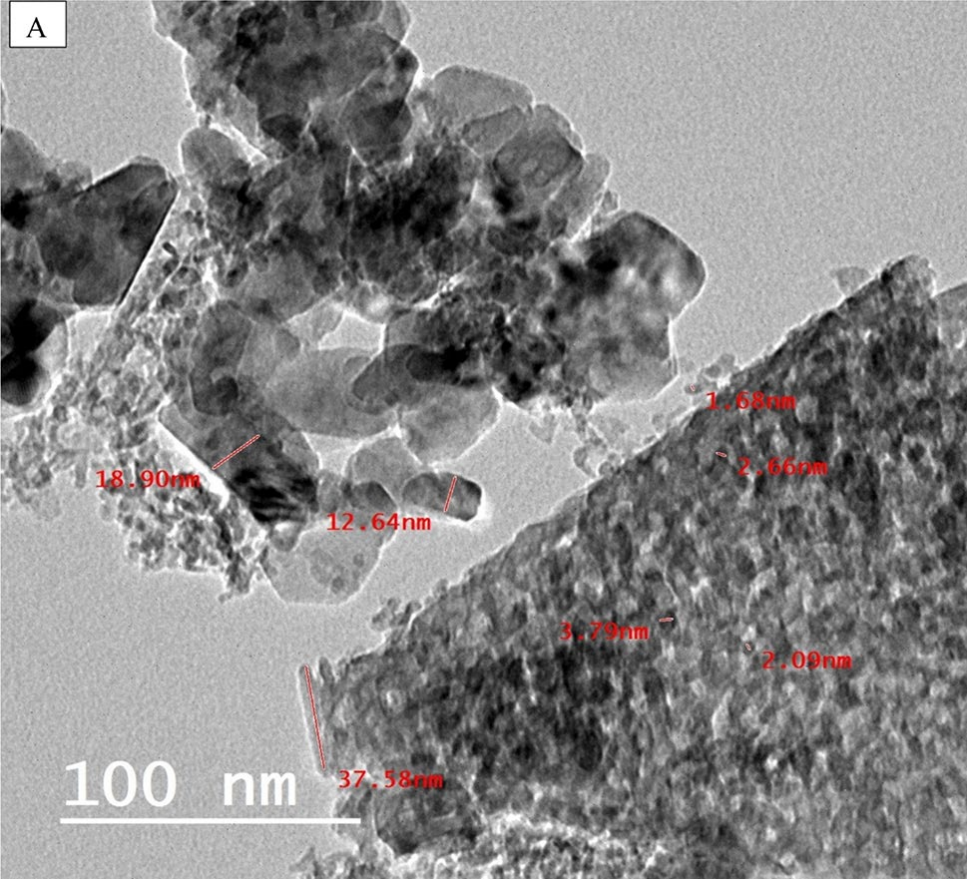

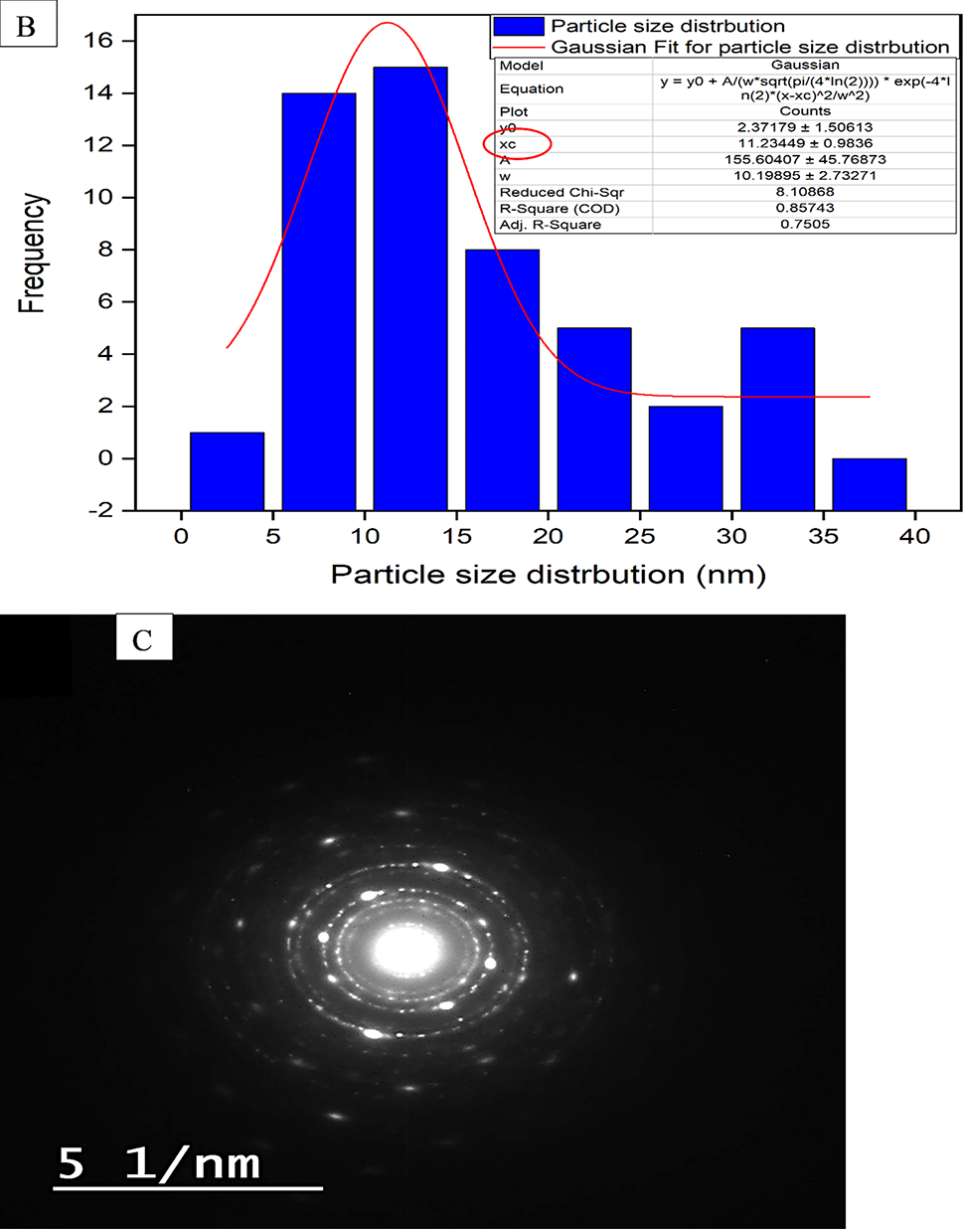
(**A**) High-resolution transmission electron microscopy, particle size distribution (**B**), and SAED images (**C**) for the nano mullite.

Another practical approach for characterizing nanomaterials is transmission electron microscopy (TEM). This quantitative method determines the nanomaterials’ particle size and shape^33^. Figure 4 (A and B) showed that the mullite nanoparticles were examined using transmission electron microscopy (TEM). The mullite nanoparticles exhibited an ideal orthorhombic crystal structure. Their particle size, as determined by a Gaussian mixture model and histogram developed with the Java 1.8.0 172 with ImageJ (1.53e) tool^29,34,35^, ranging from 5 to 40 nm with an average particle size of 11.2 nm, as shown in Fig. (4B). This finding is consistent with the results obtained from XRD analyses, which indicated an average crystallite size of 9.9 nm.

In conjunction with SAED, the HR-TEM revealed successive concentric rings with a well-organized arrangement of lattice edges. This confirmed the excellent crystallinity and typical polycrystalline state of the nano mullite, as depicted in Fig. (4 C)^30,36^.

### Brunauer-Emmett-Teller (BET) analysis

According to nitrogen adsorption test (BET) results, the synthesized nano-mullite displayed a type IV-(a)-H3 hysteresis loop isotherm, as classified by IUPAC-1985 for physisorption isotherms, with a notable rise in the adsorbed volume beginning at P/P_0_ = 0.877, as indicated by the BET study’s N_2_ adsorption-desorption measures supplementary Fig. (1), mullite nanoparticles have an average pore size of 8.329 nm, a surface area of 85.78 m^2^ /g, and a pore volume of roughly 0.516 cm^3^ /g, indicating an acceptable surface area and porosity at the mesoscale of the synthesized nano- mullite^30,37–39^.

### Contact angle

Contact angle measures have been performed to assess the hydrophilicity and hydrophobicity of mullite nanoparticles (Supplementary Fig. 2). The average contact angle for the nano mullite is known to be 115.3°. The value is much greater than 90° indicating that the synthesized nano mullite^31,40^, possesses hydrophobic qualities. This makes it well-suited for absorbing organic molecules through hydrophobic interactions and Van der Waals’ forces. Also, this hydrophobic nature enables the nano mullite to effortlessly float on the surface of the water, facilitating its convenient collection^41–43^, examples of hydrophobic materials applied in dyes removal such as boron nitride (BN) nanosheets for removal of congo red^43^, hydrophobic silica aerogel for methylene blue removal^41^ and sodium dodecyl sulfate modified nanocluster (Al_30_) coagulum for sudan(III) removal^44^.

The characterization of nano mullite reveals its significant attributes, including a substantial surface area, hydrophobic nature, and mesoporous structure. These properties make it suitable for many applications, such as serving as an adsorbent, catalyst, and sensor and facilitating the removal of different organic dyes. The synthesized nano mullite can be utilized for the adsorption removal of cresyl fast violet in aqueous solutions.

### Zeta potential study

Zeta potential (ZP) is a physical-chemical property that examines the stability of colloidal particle systems^45^. Electrophoretic mobility (EZP) measurements include passing an electrical field through the sample to track the movement of the nanoparticles. It is generally accepted that nanoparticles having zeta potentials between − 10 and + 10 mV are neutral. Nonetheless, strongly cationic and anionic nanoparticles are defined as having zeta potentials over + 30 mV and below − 30 mV, respectively. The calculation of zeta potential is determined using the Henry equation^46^. The zeta potential analysis was conducted on nano-mullite, revealing a distinct peak at (-17.8) mV. This negative value demonstrated the anionic nature of the nano-mullite, as depicted in Supplementary Fig. (3). The anionic nature of nano mullite allows it to easily absorb a cationic dye like cresyl fast violet by its electrostatic attraction. This attraction occurred between the surface of anionic nano mullite and the positive charge of the dye (^+^NH_2_R group).

### Statistical methods and data analysis

Within the removal analysis, depending on adsorption (RDA), the parameters are measured as a percentage of removal, so a mathematical model must be fitted to the data. The software construct Expert 7 was utilized to construct the tests and create a correlation model for the elimination percentage. A regression equation was created to model the output reactions based on three input parameters: pH, adsorbent amount, and contact time. Several tests have been conducted, manipulating various elements to achieve a more precise and accurate outcome. The model’s fit has been assessed using ANOVA to determine its effectiveness. In addition, some tests were conducted to determine the importance of the model. The ANOVA analysis for removal based on adsorption is presented in Table 1. The study has a confidence level of 95%. The notation ‘*P* < 0.05’ indicated the significance level for each term in the model. Table 1 demonstrated a strong alignment between the model and the experiment results. The R_2_ and Adj R_2_ values, representing the coefficient of determination, were recorded to exceed 95%. If the R_2_ value equals one, the model accurately represents the actual statistics. The insignificance of ‘lack of fit’ is a crucial factor in model selection^37^. The Model F-value of 50.22 proved that the model is statistically significant. The probability of a “Model F-Value” being significant is only 0.01%, which suggested that it is likely due to noise. Model terms are significant when the “Prob > F” values are below 0.0500. The model terms A, B, AB, A_2_, and B_2_ are considered significant in this scenario. The “Pred R-Squared” value of 0.8833 is reasonably consistent with the “Adj R-Squared” value of 0.9115.

**Fig. 5:**
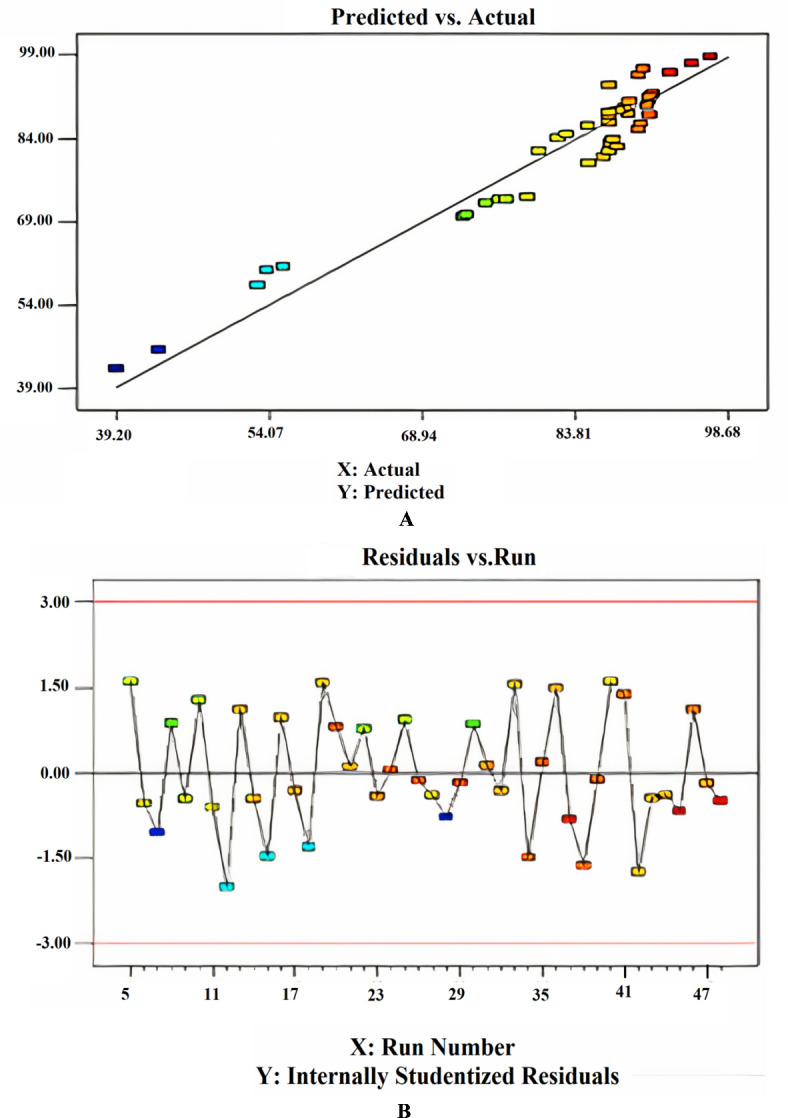

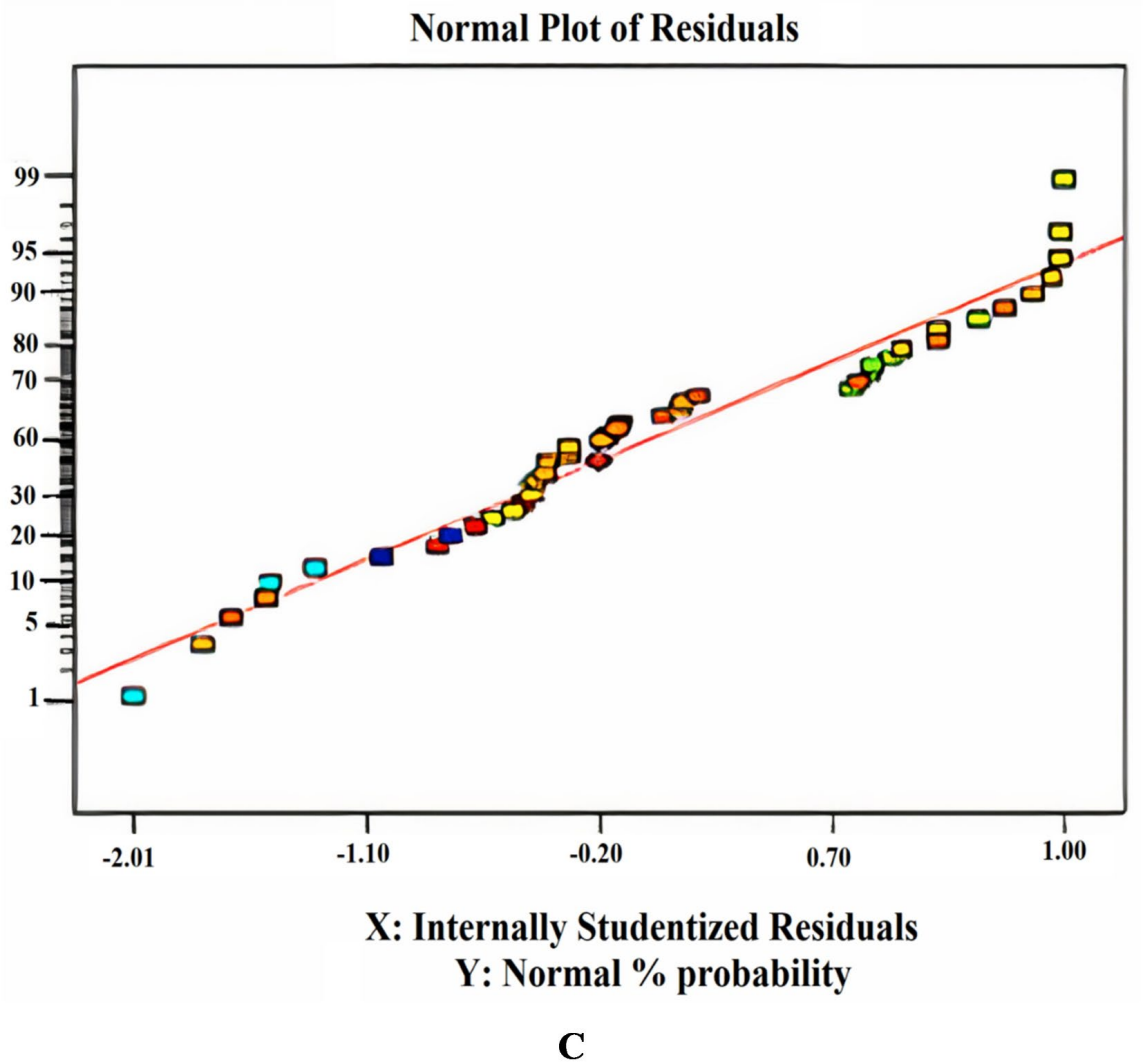
Predicted against the actual values (**A**), randomized distribution of error (**B**) and normalization plot (**C**).

**Fig. 6:**
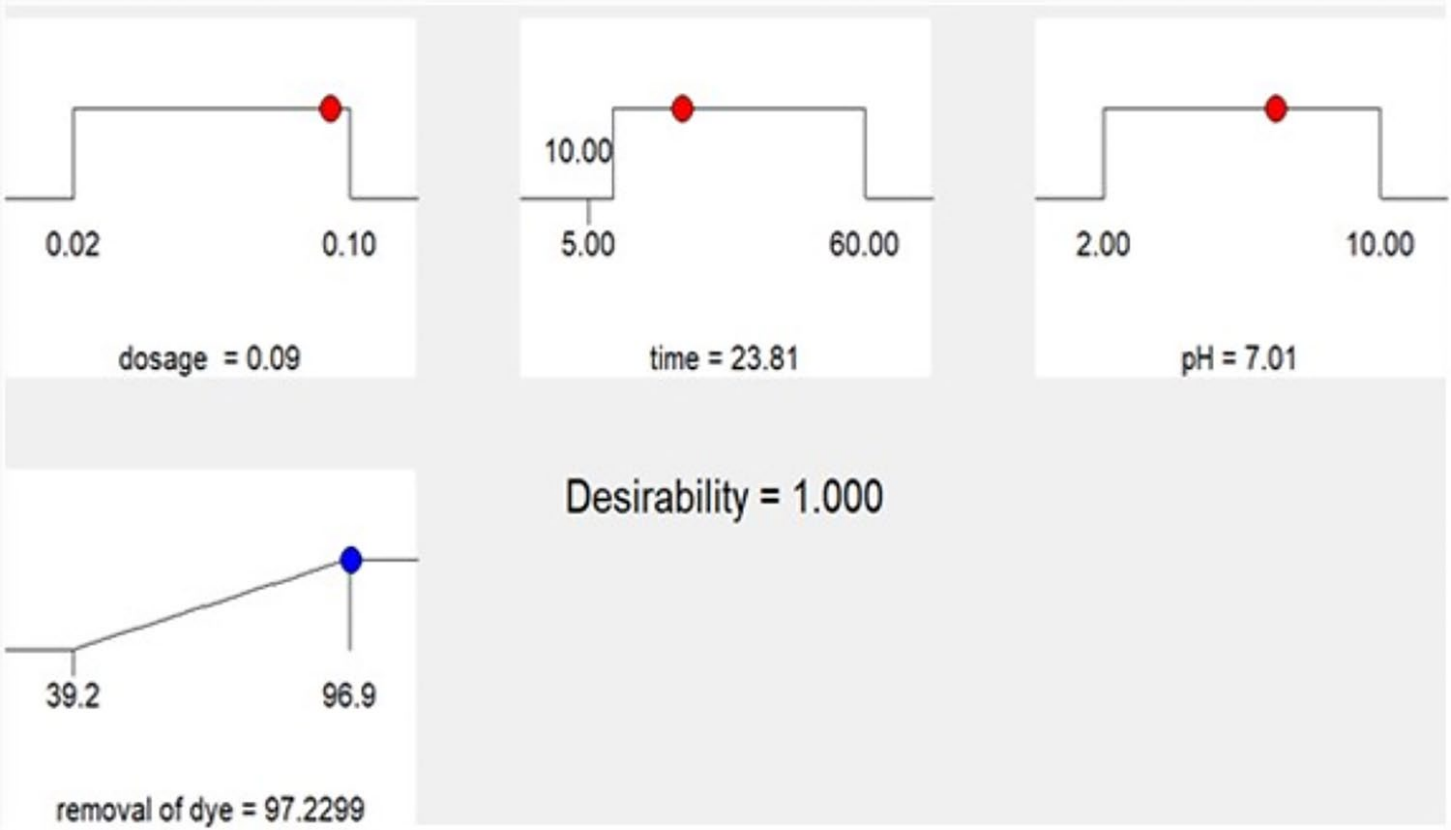
Ramp plot with minimum dosage amount and contact time corresponding to the highest dye removal with maximum desirability.

**Fig. 7:**
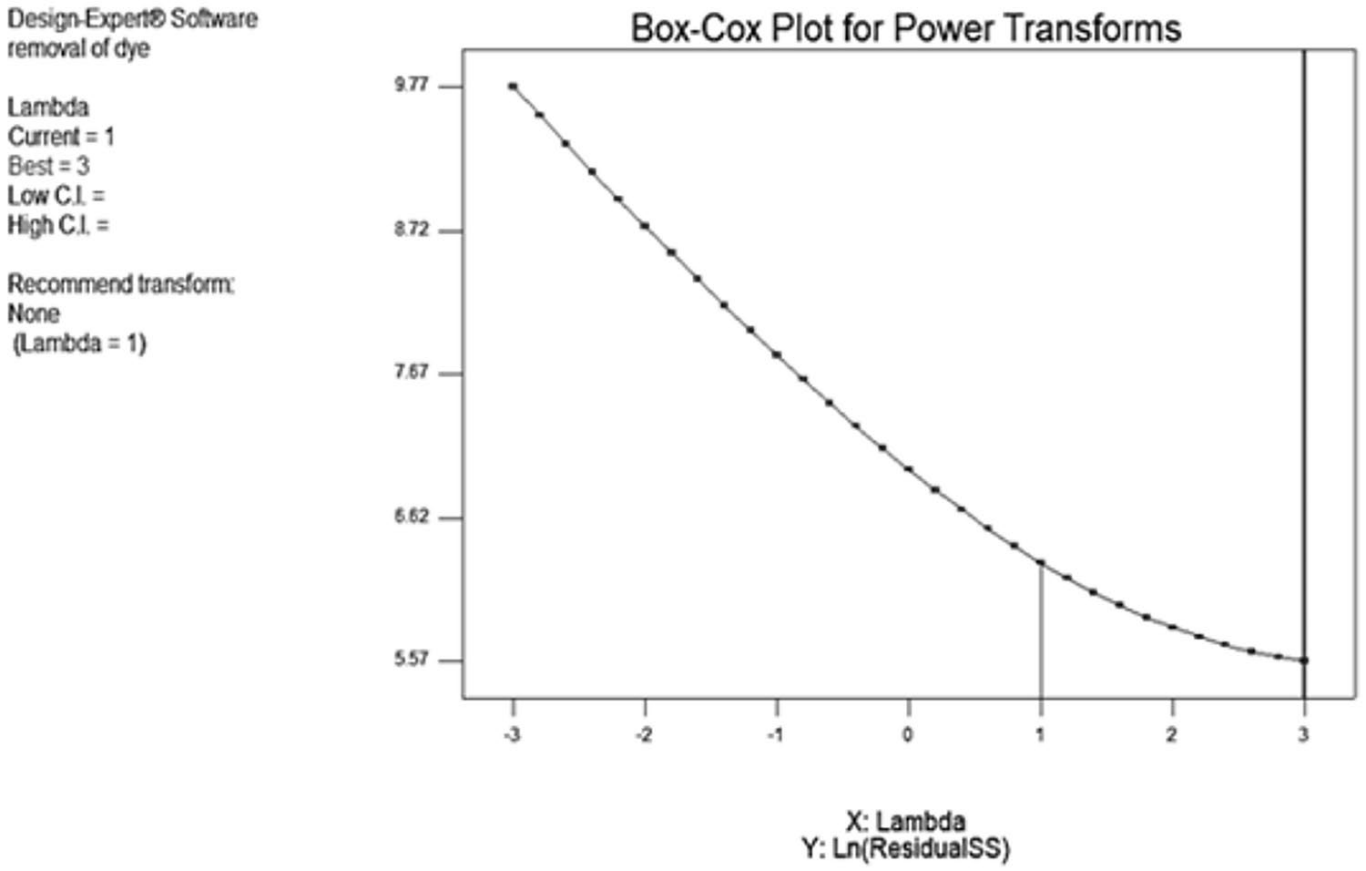
Coxbox plot for RDA model with unnecessary for power transformation.

**Fig. 8:**
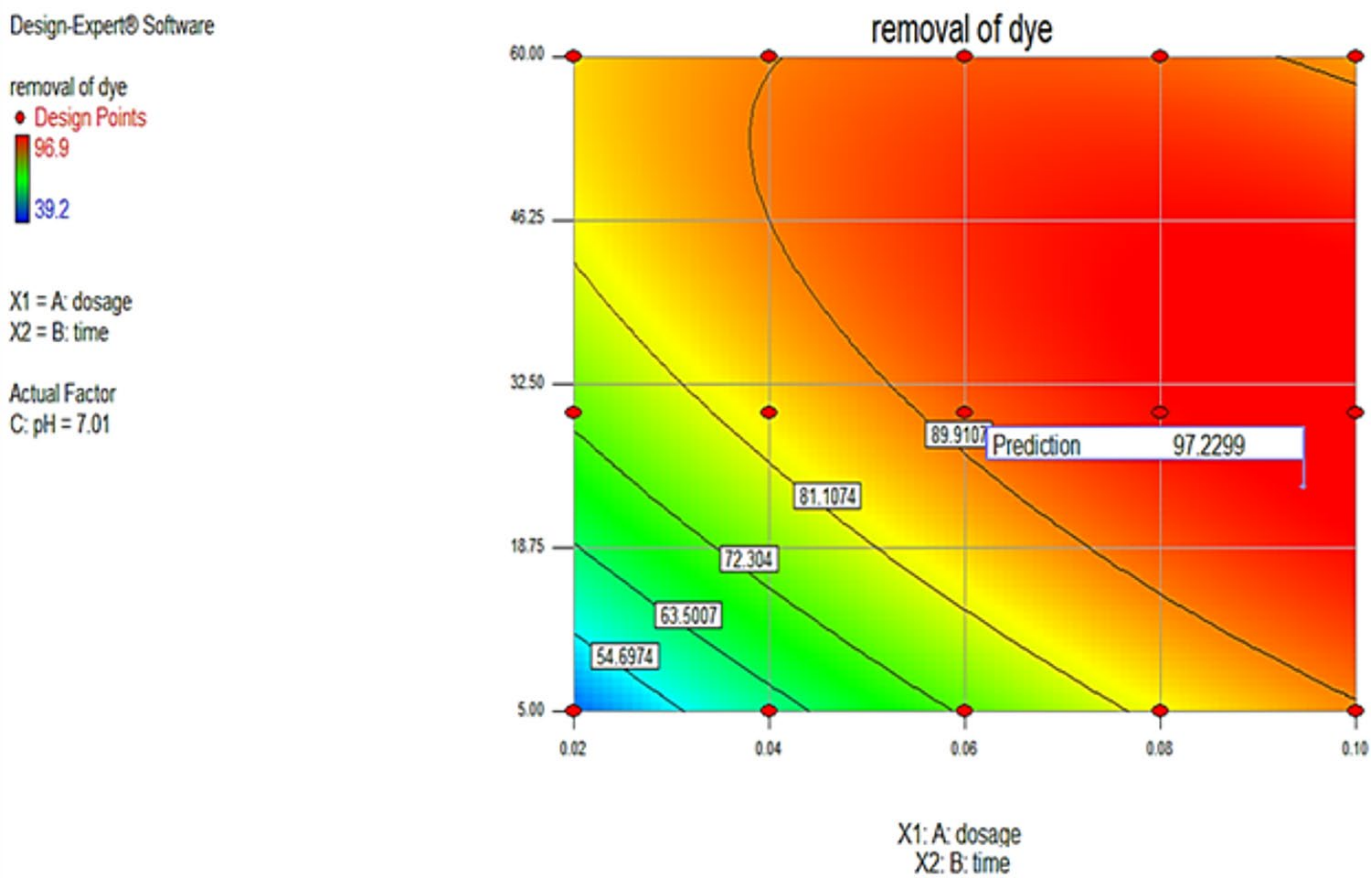
Contour graph for different parameters with maximum prediction.

**Fig. 9:**
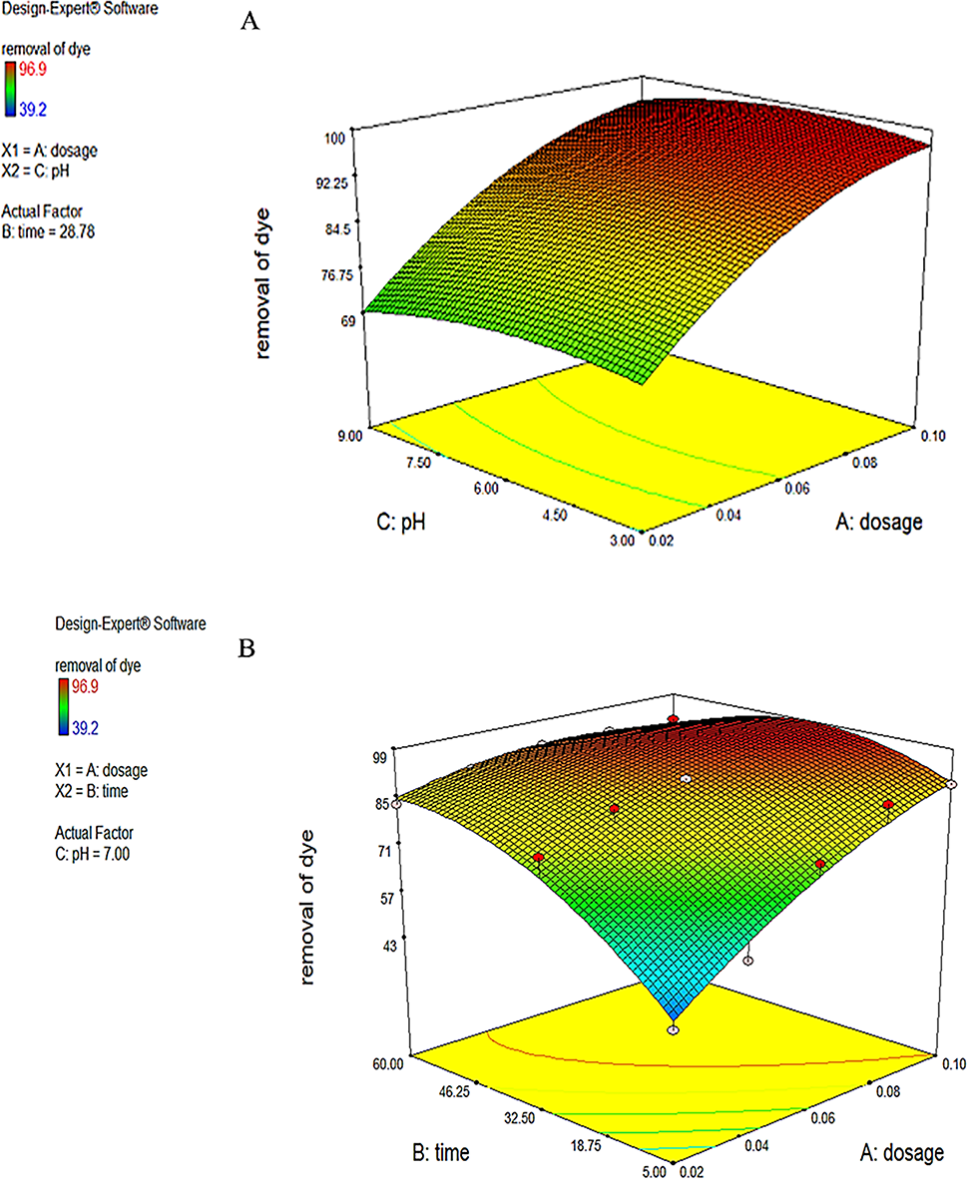
3D curves of the removal of dye different content of adsorbent versus pH (**a**), or contact time(**b**).

“Adeq Precision” quantifies the ratio of signal to noise. A ratio over 4 is preferable. The ratio of 29.549 signifies a satisfactory signal. This paradigm is applicable for navigating the design space. The appropriateness of the derived model has been assessed using a probability plot. Figure (5a) demonstrates that the observed and forecasted values are statistically similar. The presence of mistakes distributed linearly suggests that the model is suitable. The relation between the projected removal percentages and the investigational fallouts is illustrated in Fig. (5b). The residuals and predicted findings demonstrated a significant correlation. In addition, Fig. (5c) illustrated that the errors exhibited a normal distribution. This is evident from the typical probability plot of residuals, which showed that the mistakes align along a linear pattern. The ideal scenario is that the standard plot of residuals follows a linear pattern, showing the absence of any anomalies. The data does not need to align precisely with the line. A practical guideline is known as the “fat pencil” test. Suppose you can completely obscure all the data points by placing a thick pencil over the line. In that case, the data may be considered adequately conforming to a normal distribution. Regarding this situation, the approach seems satisfactory. Figure (6) illustrates the optimizer factors with a high attractiveness, reaching a value of 1.00. Figure (7) illustrates the Cox box plot, which provides evidence for the adequacy of the model without requiring any power transformation. Figures (8 and 9) illustrated the contour plot and 3D plots that depict the impact of nanomaterial dosage on pH and contact time.

**Table 1:** ANOVA for response with analysis of variables affecting the efficiency of dye removal.

Source		Sum of Squares		Df		Mean Square		F value		*p*-value Prope > F			
Model		7174.81		9		797.20		50.22		< 0.0001		Significant	
A-dosage		2729.78		1		2729.78		171.96		< 0.0001			
B-time		2421.10		1		2421.10		152.51		< 0.0001			
C-pH		32.12		1		32.12		2.02		0.1640			
AB		1352.84		1		1352.84		85.22		< 0.0001			
AC		1.30		1		1.30		0.082		0.7755			
BC		0.87		1		0.87		0.055		0.8160			
A^2^		218.60		1		218.60		13.77		0.0007			
B^2^		952.50		1		952.50		60.00		< 0.0001			
C^2^		50.50		1		50.50		3.18		0.0834			
Residual		539.74		34		15.87							
Cor total		7714.55		43									
Std. deviation		3.98				R-Squared		0.9300					
Mean		82.26				Adj R-Squared		0.9115					
C.V. %		4.84				Pred R-Squared		0.8833					
PRESS		900.02				Adeq. Precision		29.549					
**Factor**	**Name**		**Level**		**Low Level**		**High Level**		**Std. Dev.**		**Coding**		
A	Dosage		0.100		0.020		0.100		0.000		Actual		
B	Time		30.00		5.00		60.00		0.000		Actual		
C	pH		7.00		3.00		9.00		0.000		Actual		
**Response**	**Prediction**		**SE Mean**		**95% CI Low**		**95% CI High**		**SE Pred**		**95% PI Low**		**95% PI High**
Removal of dye	98.6699		1.74		95.14		102.20		4.35		89.84		107.50

## Cresyl fast violet removal by using the batch method

The impact of pH, temperature, stirring rate, adsorbent dosage, contact time, and initial dye concentration have all been studied concerning dye removal to achieve practical and ideal parameters showing the most excellent removal efficiency.

### pH effect

pH is a crucial operating parameter that influences the charges on the surface of the adsorbent in addition to interfacial transport phenomena to anticipate the adsorption process^47,48^. Using the pH scale, an aqueous solution is classified as essential (pH more than 7) or acidic (pH less than 7). The pH of the media affects how many electrostatic charges the ionized dye molecules transfer. The pH of an aqueous medium will so affect the rate of adsorption^49^.

**Fig. 10:**
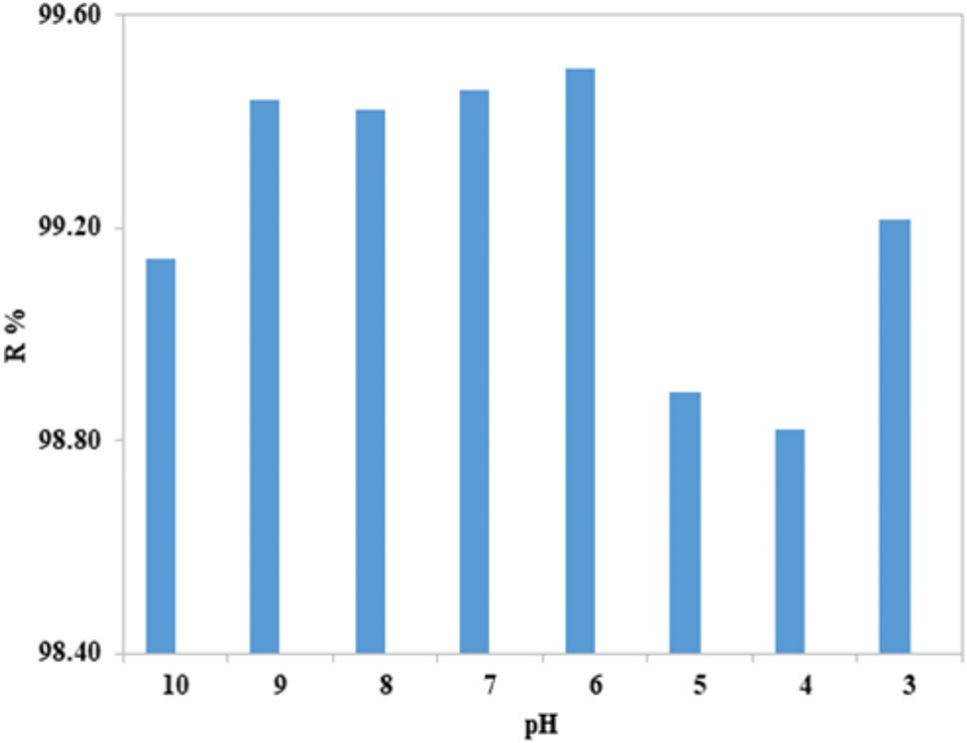
The effect of pH on the removal efficiency.

To achieve the highest level of dye removal, the adsorption of dyes on the relevant adsorbent materials was studied at different pH levels (3–10), using a dose of 0.1 g of nano mullite in 100 ml of cresyl fast violet (CFV) dye (10 ppm). HCl or NaOH were used to adjust the pH. As shown in Fig. (10), The most significant elimination effectiveness was at pH = 6 and 7, and the removal was nearly the same, which equals 99.50%. The pH was about 7 once mixing absorbent and adsorbate gave the maximum removal %. So, the adsorption process can be done without any modification for pH.

### Adsorbent dose effect

**Fig. 11:**
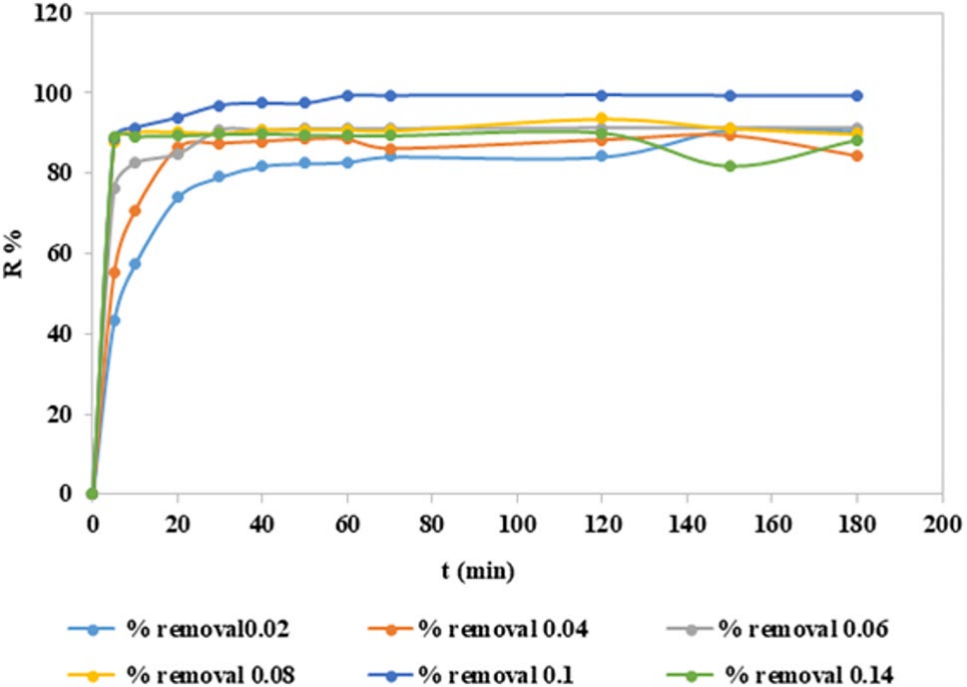
Elimination efficiency with different dosages of nano mullite (g) versus time.

The effect of adsorbent dosage on adsorption may be tested by preparing an absorbent-adsorbate solution with different quantities of adsorbents added to a predetermined starting dye concentration. Because there are more sorption sites at the adsorbent surface as the dosage of the adsorbent is raised, the percentage of dye removal usually increases as well^50^ the removal efficiency increased to 99.50%. The adsorbent dosage effect has been studied by changing the amount of nano mullite from 0.02 g to 0.14 g into 100 ml of CFV dye (concentration 10 ppm) at pH = 7, as declared in Fig. (11). The removal percentage is more than 90% since the adsorbent concentration is more than 60 mg.

### Agitation speed effect

**Fig. 12:**
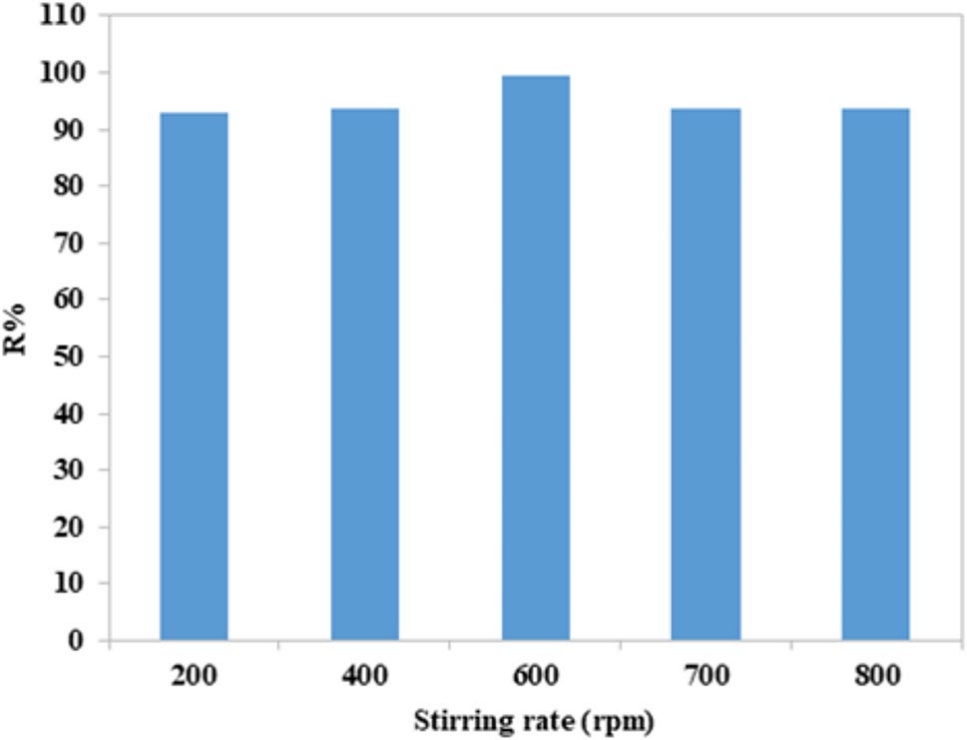
The stirring rate factor with removal percentage.

The dye was removed at different stirring rates (from 200 to 800 rpm), 100 ml of CFV dye (10 ppm) with a dose of 0.1 g of nano mullite, pH = 7. The removal rate rises from 92.47% to its optimum point of 99.50%. This result may happen as the stirring increases kinetic energy between molecules, which increases the collision and lessens the resistance of the boundary layer. Furthermore, increasing the agitation speed promotes intimate contact between the adsorbent and adsorbate phases by lessening the effect of external mass transfer^51^. As shown in Fig. (12), agitation speed doesn’t significantly affect the adsorption rate. The removal percentage is more than 90% for all, but the best removal rate was 99.50% at 600 rpm.

### Contact time effect

**Fig. 13:**
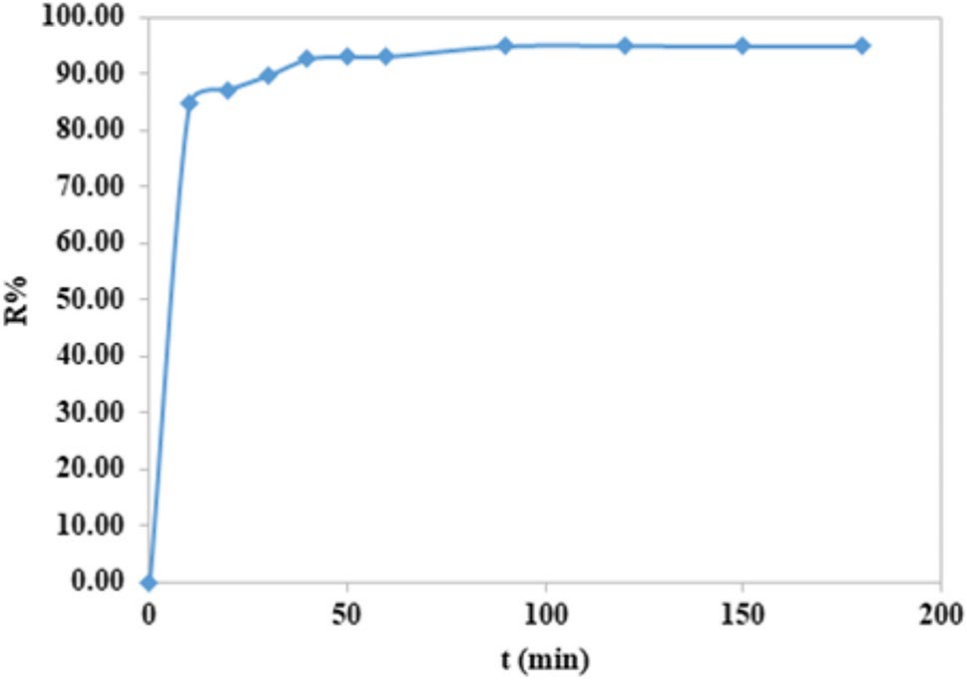
Contact time effect on the removal of CFV dye with nano mullite.

At room temperature, the time taken for removal of CFV using nano-mullite for the adsorbent dose showed fast adsorption of the dye (removal % = 89) in the first 5 min by keeping the other factors (pH, rpm, dosage, dye concentration) constant. After that, the adsorption capacity rises with time and eventually keeps constant at 60 min; at this time, no more dye will be removed from the solution, and the amount of dye being adsorbed onto the material and the amount of dye desorbed from the adsorbent is in a condition of dynamic equilibrium as shown in Fig. (13). The % removal was more than 90% after 10 min, but after 30 min the removal of dye gives its maximum.

### Concentration effect

**Fig. 14:**
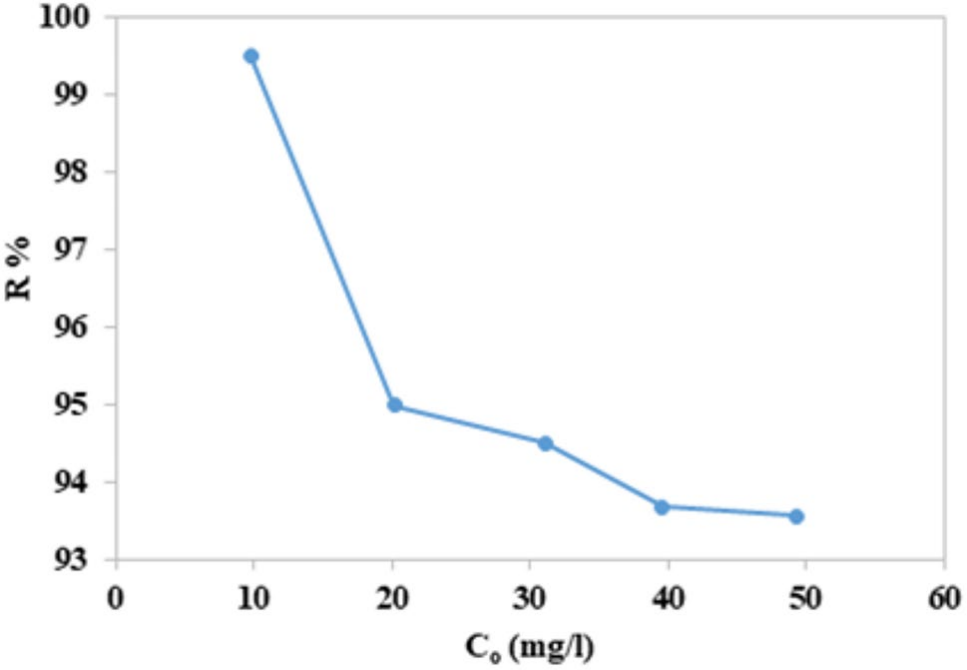
Removal efficiency of different concentrations of CFV dye with nano-mullite.

Dye adsorption, a mass exchange process, is often defined as a collection of dye at the interface of the liquid-solid phase. The push required to overcome the mass transfer barrier from the aqueous phase to the solid state is provided by the initial concentration of the dye. As initial concentration increases, the driving force gets better^52^. The impact of the dye concentration was examined while maintaining the other factors constant, as shown in Fig. (14). It was observed that by increasing the initial concentration from 10 mg/l to 20 mg/l and keeping the ideal content of each parameter, the removal decreased from 99.46 to 95%. Increasing the concentration to more than 20 mg/l, the removal was still approximately 94% to 50 mg/l. This reflects the high surface area of the nano-mullite and its ability to remove high concentrations of dye with high efficiency.

## Adsorption kinetics

The kinetic models of CFV dye removal by nano mullite have been studied using pseudo-first-order, pseudo-second-order and Intra-particle diffusion.

### The pseudo-first order and pseudo-second order

Equations for adsorption kinetics are as follows:

1 $$\:\text{log}\left({\text{q}}_{\text{e}}-{\text{q}}_{\text{t}}\right)=\text{log}{\text{q}}_{\text{e}}-\left(\frac{{\text{k}}_{1}}{2\cdot\:303}\right)\text{t}$$

2 $$\:\frac{\text{t}}{{\text{q}}_{\text{t}}}=\left(\frac{1}{{\text{k}}_{2}{\text{q}}_{\text{e}}^{2}}\right)+\left(\frac{1}{{\text{q}}_{\text{e}}}\right)\text{t}$$

The pseudo-first order and the pseudo-second order, respectively, are by Eqs. 1 and 2.

K_1_ (min^−1^) and k_2_ (g/mg. min) are the adsorption rate constants of the Pseudo-first and Pseudo-second order expressions. Also, q_e_ q_t_ the quantities of CFV adsorbed at equilibrium and after time t in (mg/g), respectively^53,54^. The pseudo-first-order module indicated the amount of adsorbate on the adsorbent surface over a certain time (t). Concentrations that considerably affect the rate of a reaction are considered consistent with it. The term referred to adsorption on heterogeneous surfaces. The pseudo-second-order module was meant to mimic the actual reaction as nearly as feasible.

**Fig. 15:**
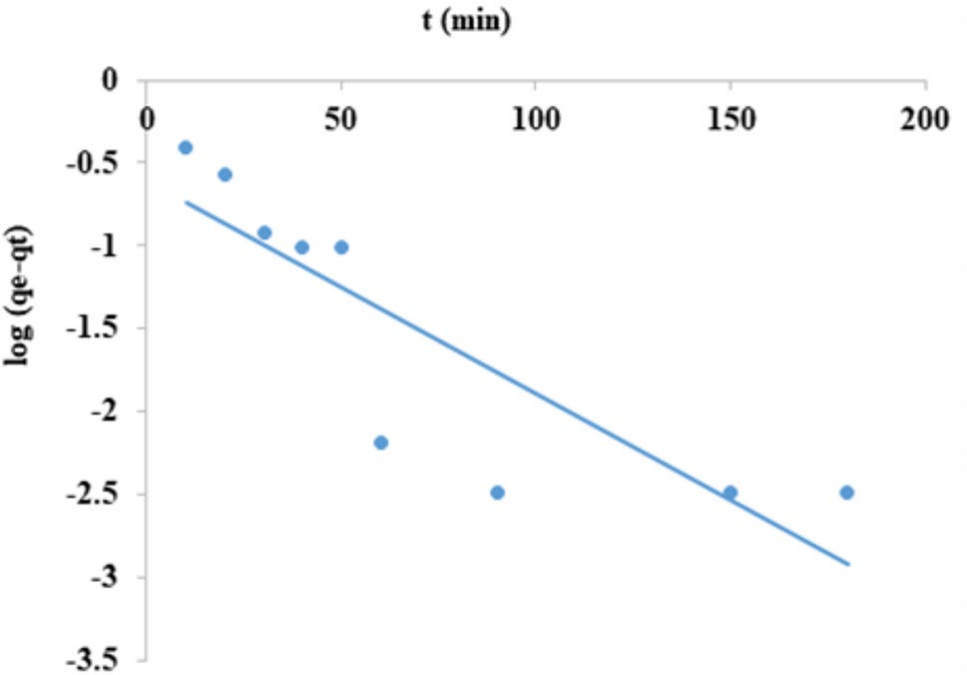
Pseudo-first order of cresyl fast violet adsorption into nano mullite.

**Fig. 16:**
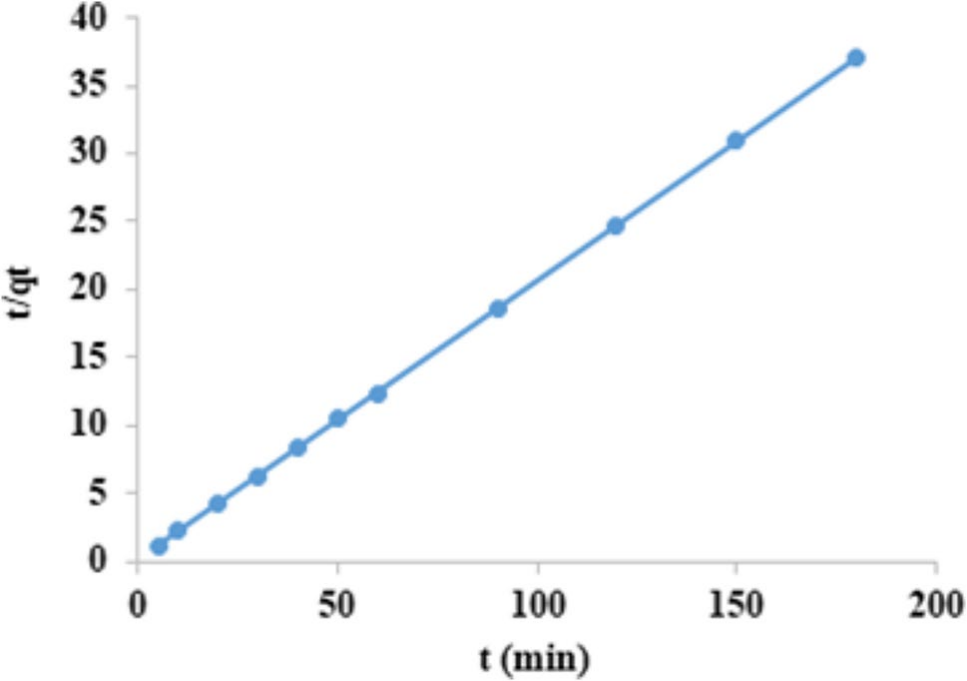
Pseudo-second order of cresyl fast violet adsorption into nano mullite.

As shown in Fig. (15) and Table (2), when log (q_e_-q_t_) against time was plotted for the Pseudo-First-order model a linear relationship was noticed with R^2^ value equal 0.739, and k_1_ = 0.022 min^−1^. However, in Fig. (16) when plotting t/q_t_ against t, a straight line comes with R^2^ equals 1, and k_2_ = 0.225 g/mg.min for the pseudo-second-order model. Also, in the case of first order model the value of q_e theoretical_ = 4.03 mg/g, q_e experimental_ = 4.85 mg/g unless in case of second order model the value of q_e theoretical_ = 4.88 mg/g, q_e experimental_ = 4.85 mg/g. Therefore, due to the closeness between the q_e_ theoretical and q_e_ experimental of the pseudo-second order, as well as the high regression R^2^ value of unity, the pseudo-second-order model is the most appropriate model for the removal of the CFV. So, the adsorption process will depend on the of nanomaterial and dye amount.

### Intra-particle diffusion

It offers a helpful tool to help comprehend the process of CFV absorption onto nano mullite. This model states that the movement of CFV within the adsorbent particles’ pores controls the adsorption rate. This model is critical for understanding the rate-determining step in liquid adsorption systems. In a batch process, the rate-determining step might be either solute diffusion from the bulk of the solution to the solid surface (intra-particle diffusion) or diffusion over the solid surface’s boundary layer (film).

Intra-particle diffusion can be calculated from the following Eq. (3):

3 $$\:{q}_{t}={k}_{i}{t}^{0.5}+C$$

q_t_ the quantities of CFV adsorbed (mg/g) at time t, $\:{k}_{i}\:$is the diffusion rate constant of intra-particle (mg/ g.min^1/2^); C is the boundary layer thickness constant (mg/g)^53,54^. If intra-particle diffusion determines the rate, the plot of q_t_ vs. t^0.5^ should be linear, intersecting the origin with a zero intercept. If there is any deviation from linearity, it is evident that the rate-determining step should be governed by boundary layer diffusion.

**Fig. 17:**
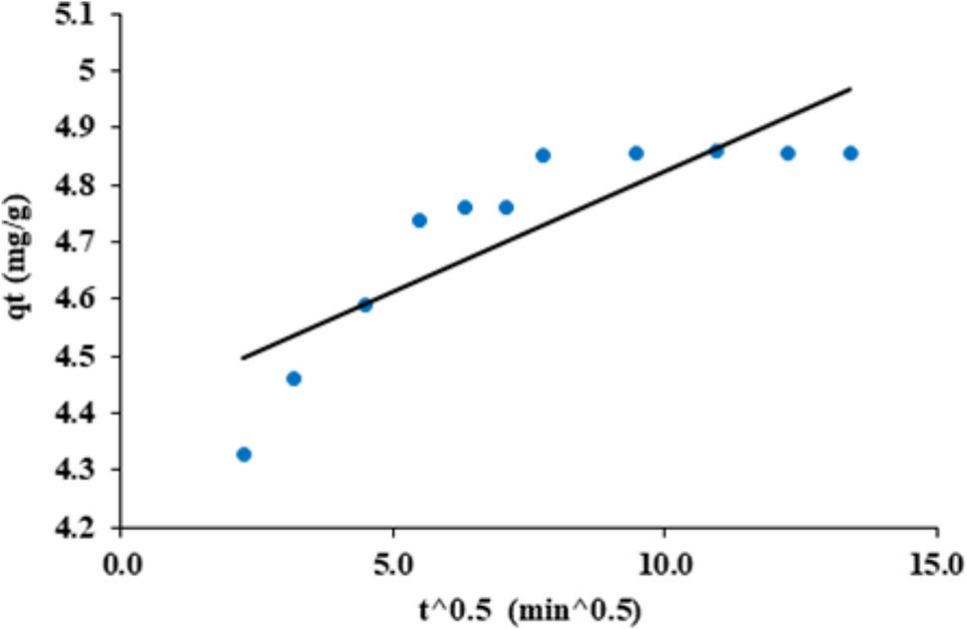
Intra-particle diffusion of cresyl fast violet adsorption into nano mullite.

As shown in Fig. (17) and Table (2), when plotting the relationship between q_t_ and t^0.5^, it should show a straight line, as predicted with k_i_ = 0.04 mg/g. min^1/2^, C = 4.40 mg/g, and R^2^ = 0.7245. It doesn’t intersect the zero point. So, the rate determining step is controlled by boundary layer (film) diffusion.

**Table 2:** Adsorption kinetics of cresyl fast violet adsorption on mullite nanoceramic binding material.

Adsorption kinetics	Results
Pseudo-first order	k_1_ = 0.03 min^−1^
	R^2^ = 0.739
	q_e_ cal = 4.03 mg/g
	q_e_ exp =4.85 mg/g
Pseudo-second order	q_e_ cal = 4.88 mg/g
	q_e_ exp =4.85 mg/g
	k_2_ = 0.225 g/mg .min
	R^2^ = 1
Intra-particle diffusion	R^2^ = 0.7245
	k_i_ = 0.04 mg/g. min^1/2^
	C = 4.40 mg/g

From Figs. (15–17) and Table 2, it was concluded that adsorption kinetics would follow pseudo-second-order kinetics as a high value of regression R^2,^ which is unity and the estimated q_e_ values obtained from this rate model exhibit a firm agreement with the experimental q_e_ values^55^.

## Adsorption Isotherm

Isotherm studies yield crucial parameters for predicting and optimizing adsorption processes by elucidating the distribution of adsorbents between the solid and solution phases at equilibrium. Adsorption isotherms reveal the behavior of adsorbents and guide their utilization. Adsorption isotherms, or the ratio of the amount absorbed to the amount in solution at a fixed temperature equilibrium, illustrate equilibrium studies that provide the absorbent and adsorbent’s ability. The Langmuir, Freundlich, Temkin, and DKR models were used to analyze and fit the experimental results.

### Langmuir isotherm

The fundamental premise of the Langmuir isotherm is that homogenous adsorption energies are transferred to the adsorbent surface. It is predicated on the assumption that monolayer adsorption takes place on an entirely homogeneous surface, has a finite number of identical sites, and has negligible interaction between adsorbed molecules^55^. The Langmuir isotherm model was expressed as (Eq. 4):^56^

4 $$\:{\text{q}}_{\text{e}}=\:{\text{q}}_{\text{m}\text{a}\text{x}}\text{*}{\text{b}\text{C}}_{\text{e}}/1+\hspace{0.17em}{\text{b}\text{C}}_{\text{e}}\:$$

by linearizing, it will be as the following (Eq. 5):

5 $$\:\frac{{c}_{e}}{{q}_{e}}=\frac{1}{{q}_{\text{m}\text{a}\text{x}}*b}+\left(\frac{1}{{q}_{\text{m}\text{a}\text{x}}}\right){c}_{e\:}\:$$

**Fig. 18:**
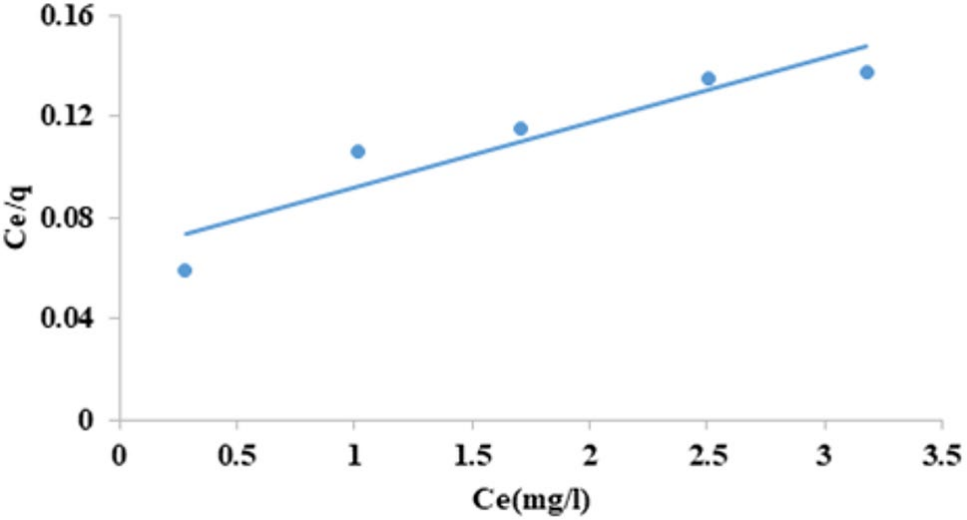
Langmuir isotherm for cresyl fast violet adsorption into nano mullite.

Where is the C_e_ equilibrium concentration in the solution (mg/L), q_e_ is the quantity of CFV sorbed per unit mass of adsorbents (mg/g), The Langmuir constants q_max_ and b correspond to the monolayer sorption capacity (mg/g) and the Langmuir equilibrium constant (L/mg), respectively. As shown in Fig. (18) and Table (3), the Langmuir isotherm plotted C_e_/q_e_ against C_e_ and indicated a straight line with a slope of 1/q_max_ and intercept equal 1/b*q_max_. Data reported in Table (3) indicated that the Langmuir constant (b) was 0.385 L/mg, with a maximal monolayer adsorption capacity (q_max_) of 39.22 mg/g and a coefficient of correlation (R^2^) of 0.8687. Calculating the dimensionless separation factor R_L_ (Eq. 6), which is derived from the Langmuir constant equilibrium (b) and the initial concentration of CFV in the solution, denoted as $C_\circ$(mg/L), can determine the favorable or unfavorable reaction processes for Langmuir^57^.

6 $$\:{R}_{L}=\frac{1}{1+bC_\circ}$$

R_L_ explains the form and favorability of the adsorption between adsorbents and CFV. The isotherm’s favorable (0 < R_L_ < 1), unfavorable (R_L_ > 1), linear (R_L_ = 1), or irreversible (R_L_ = 0) shape can all be determined by looking at the value of R_L_^58^. The R_L_ constant value determined for the adsorption of CFV by nano mullite was 0.206, indicating that the adsorption process is favorable.

### Freundlich model

A scientific equation known as the Freundlich model is derived from the absorption of various affinities that support sites of heterogeneous surface or water. It is hypothesized that the more powerful binding sites are initially filled and that the strength of binding declines as the site becomes more populated. The expression of the model might be displayed as (Eq. 7):

7 $$\:{q}_{e}={K}_{f}{\text{C}}_{\text{e}}^{1/\text{n}}$$

**Fig. 19:**
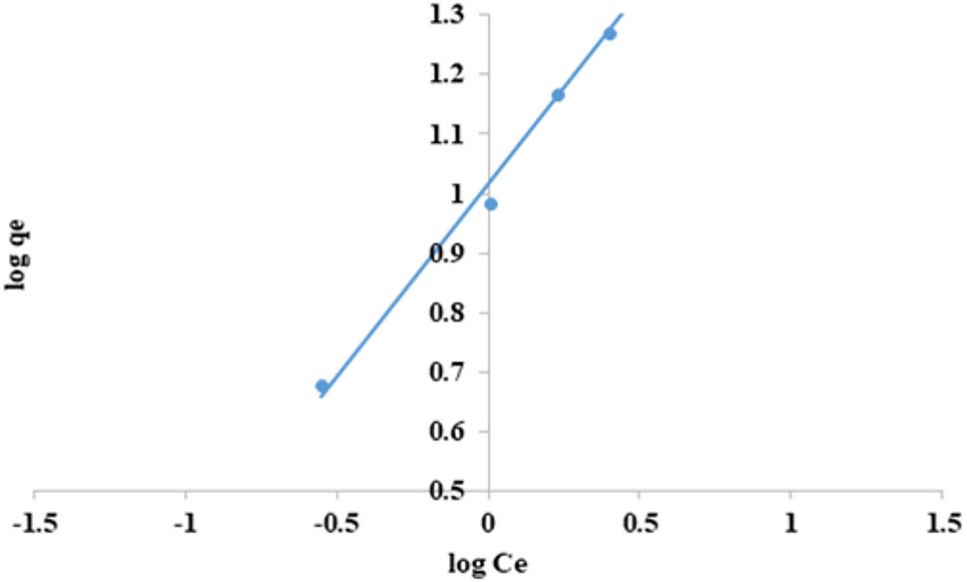
Freundlich isotherm for cresyl fast violet adsorption into nano mullite.

This expression can be rendered linear by applying the subsequent logarithms and constants (Eq. 8):

8 $$\:{log}{q}_{e}=log{k}_{f}+\frac{1}{n}{log}{C}_{e}$$

where C_e_ is The concentration of the adsorbate at equilibrium (mg L^−1^), The variable “q_e_” represents the quantity of adsorbate that is adsorbed (mg/g) K_f_ (mg/g) (L/g)^1/*n*^ and n represents the Freundlich constant, which respectively indicates the adsorption capacity and intensity of adsorption^59^. As shown in Fig. (19) and Table (3), the data was applied, and a straight line was obtained when the Freundlich isotherm was plotted (log q against log C_e)_ and gave K_f_=10.35 (mg/g) (L/g)^1/*n*^, *n* = 1.53 and R^2^ = 0.9928. It was noted that the value of n is more than 1, indicating the physical process is favorable because if n is less than one, it indicates that the adsorption process is chemical. However, if the value of n is more significant than one, adsorption is a physical process^55,60^.

### Dubinin-Kaganer-Raduskevich (DKR)

The Dubinin-Radushkevich isotherm is an empirical model and more comprehensive than the Langmuir isotherm as it does not make assumptions about a uniform surface or consistent adsorption potential^55^. It describes the adsorption of CFV on macroporous substances, explicitly focusing on pore-filling. It distinguishes between physical and chemical adsorption to release a molecule from its location in the sorption space to an infinite distance^61,62^. The isotherm has the following linear form and is used to calculate the apparent energy of CFV adsorption onto adsorbent (Eqs. 9 and 10)^63^.

9 $$\:{ln}{q}_{e}={ln}{q}_{s}-\beta\:{\epsilon\:}^{2}$$

10 $$\:\epsilon\:=RT{ln}\left(1+\frac{1}{{c}_{e}}\right)$$

where ε denotes the Polanyi potential, q_max_ denotes the sorption capacity (mg/g). β estimates The average free energy (E) (kJ/mol) of sorption for each sorbate molecule moved from infinity in the solution to the surface of the solid as shown in the following equation: 11^64^.

11 $$\:E=\frac{1}{\sqrt{2\beta\:}}$$

**Fig. 20:**
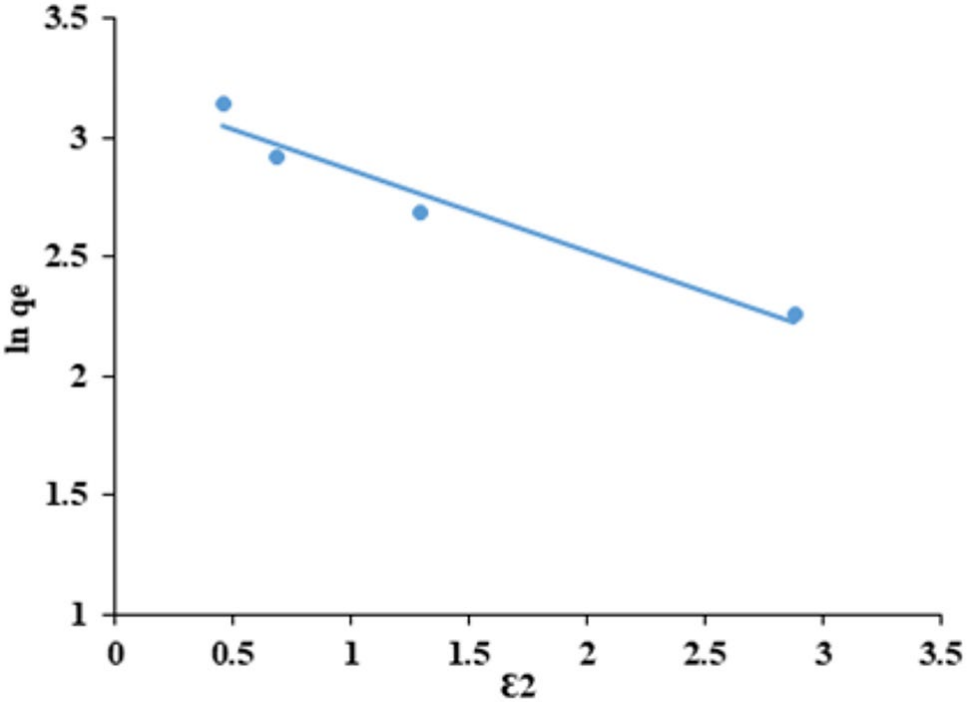
Dubinin-Kaganer-Raduskevich isotherm cresyl fast violet adsorption into nano mullite.

The type of adsorption process is mostly dependent on the value of the mean free energy (E), if $\:8<E<16$ kJ/mol the adsorption process is ion exchange, if $\:E<8$ kJ/mol, the adsorption process is physical interaction, if $\:E>16$ kJ/mol the adsorption process is chemical interaction. The value of E is 1.22 kJ/mol, so the adsorption process is physical interaction. As shown in Fig. (20) and Table (3), the previous data was applied, and straight lines were obtained when the DKR isotherm was plotted ln q_e_ against ɛ^2^ with E = 1.22 kJ/mol, q_s_ = 24.53 mg/g and R^2^ = 0.9585. The outcome of the mean adsorption energy derived from the Dubinin-Radushkevich isotherm reveals that the adsorption mechanism is primarily physisorption rather than chemisorption. This is indicated by an E value below 8 kJ/mol, which signifies that physisorption controls the adsorption process. However, if the E value falls between 8 and 16 kJ/mol, it suggests the involvement of a chemisorption process^57,61^.

### Temkin isotherm

The Temkin isotherm equation postulates that the heat of adsorption for each molecule in the layer declines linearly as the coverage increases, owing to interactions among the adsorbent and adsorbate. Additionally, it assumes that the adsorption process is characterized by a uniform distribution of binding energies up to reaching a specific maximal energy of binding (Eq. 12)^65^.

12 $$\:{q}_{e}=\text{B}{ln}A+\text{B}{ln}{C}_{e}$$

**Fig. 21:**
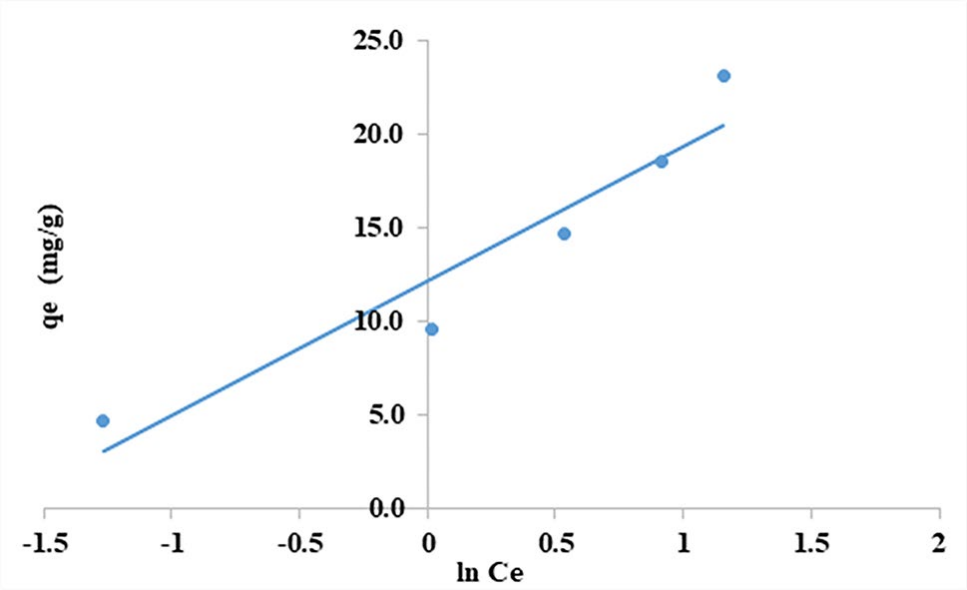
Temkin isotherm for cresyl fast violet adsorption into nano mullite.

The equilibrium binding constant, A (L/mg), represents the maximum binding energy. B = RT/b was obtained from the Temkin plot (q_e_ versus ln C_e_) where **R** = universal gas constant (8.314 J.mol) and ***T*** is the temperature (K) and **b** constant correlated to the heat of sorption (KJ/mol)^53^. As shown in Fig. (21) and Table (3), The data was applied, and Straight lines were obtained when the Temkin isotherm was plotted (q_e_ against ln C_e)_ and gave b = 0.3 kJ/mol, A = 5.47 L/mg and R^2^ = 0.9108. The lower value of b implies a weak interaction between CFV and nano mullite. Therefore, the adsorption mechanism of CFV onto nano mullite can be described as physisorption. This is because the bonding energy range for the ion-exchange mechanism is documented to be between 8 and 16 kJ/mol. In contrast, physisorption processes have adsorption energies lower than this range^55^.

Based on the analysis of the isotherm’s experimental data (Table 3), the Langmuir equation provides the least accurate fit, as indicated by the lowest correlation coefficient value of 0.8687. Nevertheless, the correlation coefficient for the Freundlich model exhibits the most outstanding value (0.9928) compared to the other models used. This suggests that the model can accurately describe the adsorption of CFV onto nano mullite.

**Table 3:** Langmuir, Freundlich, DKR, Temkin and R^2^ values of cresyl fast violet adsorption on nano mullite.

Isotherm	Results
Langmuir isotherm	q_max_ = 39.22 mg/g
	b = 0.385 L/mg
	R_L_= 0.206
	R^2^ = 0.8687
Freundlich isotherm	K_f_ = 10.35 (mg/g) (L/g)^1/*n*^
	*n* = 1.53
	R^2^ = 0.9928
DKR isotherm	E = 1.22 kJ/mol
	q_s_ = 24.53 mg/g
	R^2^ = 0.9585
Temkin isotherm	b = 0.3 0.3 kJ/mol
	A = 5.47 L/mg
	R^2^ = 0.9108

## Thermodynamic study

Thermodynamic parameters are utilized to assess the spontaneity of an absorption process. The changes in free energy (ΔG°), enthalpy (ΔH°), and entropy (ΔS°) of the absorption process were determined using the following Eqs. (13–15):

13 $$\:{K}_{d}={q}_{e}/{C}_{e}$$

14 $$\:{\Delta\:}\text{G}^\circ\:=-RTln{K}_{d}$$

15 $$\:ln{K}_{d}=-(\varDelta\:H^\circ\:)/RT+(\varDelta\:S^\circ\:)/R$$

q_e_ represents the equilibrium concentration of adsorbed dye on the adsorbent, while C_e_ represents the equilibrium concentration of the residual dye in the solution. A linear relationship is observed in a Van’t Hoff plot of the ln k_d_ vs. the reciprocal of temperature (1/T)) Supplementary Fig. 4). The values of ΔH° and ΔS° can be calculated by calculating the slope and intercept of the line. The ΔH° value of 10.17 kJ/mol demonstrates that the absorption of CFV onto nano mullite is an endothermic process. The positive ΔS° value of 0.065 kJ/mol.K indicates an increase in randomness at the absorption/solution interface during the absorption of the dye onto nano mullite. The ΔG° values at different temperatures 298, 308, and 318 K were − 2.35, -3.15, and − 3.54 kJ/mol, respectively, which indicate that CFV absorption is a spontaneous process^57,62^.

## Characterization of the nano mullite after adsorption process

**Fig. 22:**
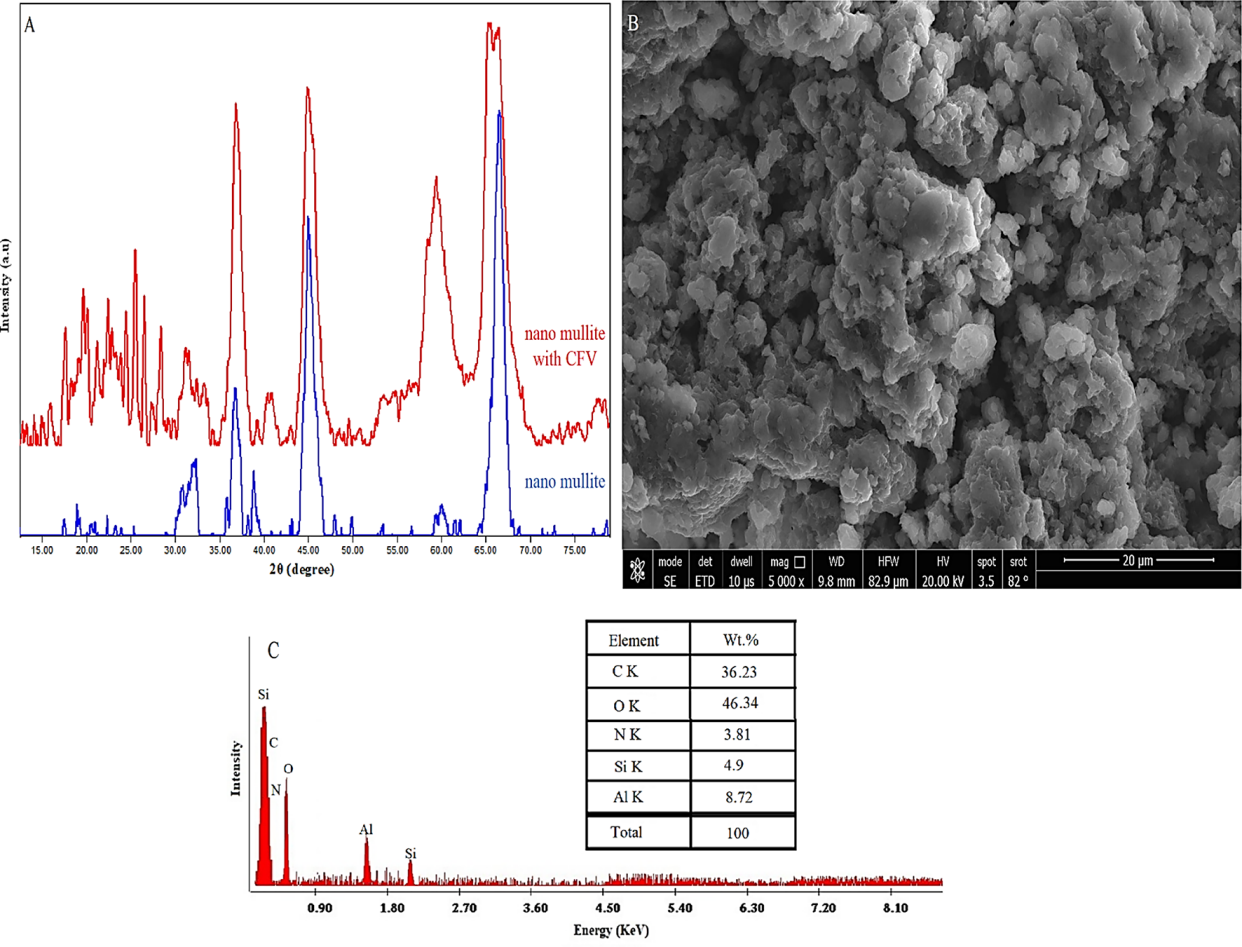
The X-ray diffraction patterns (**A**), SEM (**B**) and EDAX analysis (**C**) for the nano mullite and nano mullite with CFV.

The adsorption of CFV dye into the nano-mullite (3Al_2_O_3_.2SiO_2_) was confirmed by XRD pattern as Fig. (22 A). XRD pattern showed a characteristic peaks of the mullite nanoparticles^29^ with other different peaks which corresponding to CFV dye. The SEM image conjugated with EDAX confirm also the adsorption of the CFV into the nano-mullite by change the surface morphology of the nano-mullite and the chemical composition of nano mullite surface by revealing the composition of the CFV into it (Fig. 22B and C). The chemical composition after adsorption comprises approximately 36.23% carbon, 3.81% nitrogen, 4.9% silica, 8.72% aluminum, and 46.34% oxygen. Also, the BET analysis supplementary Fig. (5) showed decrease in the pore size and surface area after adsorption by decreasing from 8.329 nm to 85.78 m^2^/g in mullite nanoparticles to 4.02 nm and 50.23 m^2^/g in the nano mullite with CFV. So, according to this result the complete adsorption of the CFV into the nano mullite particles was confirmed.

## Desorption and reusability

**Fig. 23:**
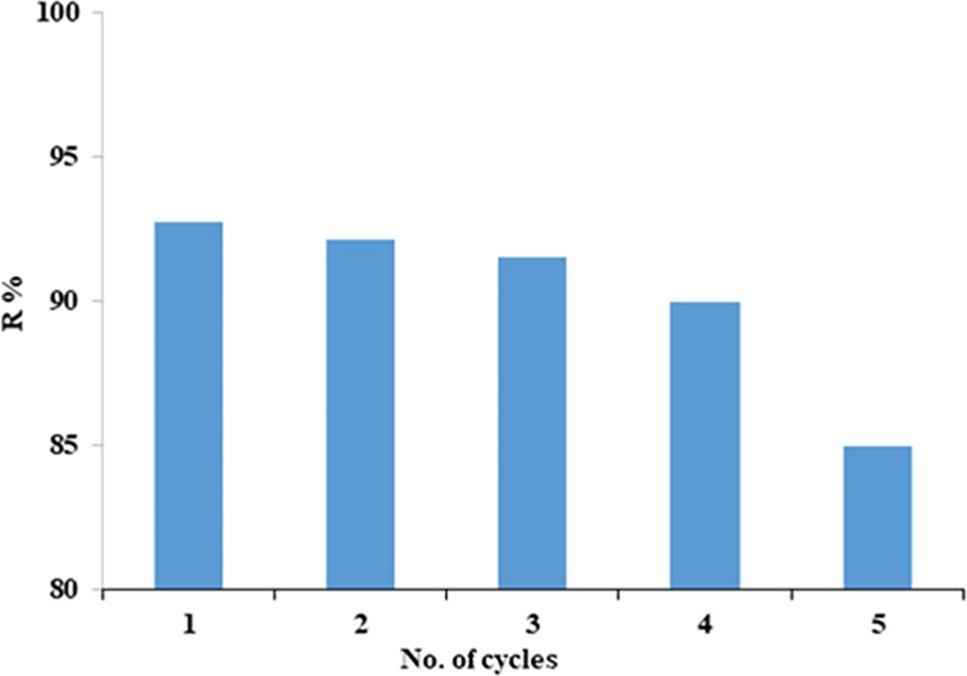
Number of cycles of reusable nano mullite for adsorption removal of CFV.

The adsorption-desorption investigations were carried out at room temperature for five cycles. The investigations were done using NaOH and HCl (1:1). CFV quantitative desorption from mullite was completed after filtering and washing with distilled water. It can be reused multiple times. Figure (23) showed that over 90% of the desorption process was effective in up to four cycles. Mullite’s reusability demonstrates the adsorbent’s affordability and usefulness, as well as its potential for evaluation and application in wastewater treatment^66^.

## Materials and experimental data

### Materials and instruments

All the chemicals, reagents, and equipment that were used are covered in supplemental data.

### Preparation of nano mullite

The mullite nanoparticle was synthesized using the same method described in our earlier study^30^. The aluminum chloride hexahydrate (AlCl_3_.6H_2_O) was dissolved in water and agitated to make a 1 M solution. Tetraethyl orthosilicate (TEOS) was mixed with water and ethanol in a 4/1 molar ratio and agitated at 80 °C for 2.5 h to hydrolyze silica. To synthesis a 3:2 molar ratio of Al_2_O_3_ to SiO_2_. The alumina chloride solution (1 M) was added to the TEOS solution and stirred for another hour. Then, the ammonium hydroxide solution was added drop by drop to maintain the blended solution’s pH at 10. Subsequently, the gel formation was subjected to a 24 h drying process at a temperature of 90 °C in an oven, followed by calcination at a temperature of 1000 °C for one hour, with a heating rate of five °C per minute. The synthesized nano-mullite underwent a range of analytical procedures, such as X-ray diffraction (XRD), scanning electron microscope (SEM) combined with energy-dispersive X-ray spectroscopy (EDAX), transmission electron microscopy (TEM), Brunauer-Emmett-Teller (BET) analysis, Zeta Potential and contact angle measurement.

### Studies on batch adsorption

The dye has been eliminated from an adsorbent utilizing the batch adsorption procedure. Batch experiments on adsorption were conducted using various amounts of adsorbent ranging from 0.02 to 0.14 g/100 ml. The temperature was kept at 25.00 ± 1.00 °C. The added cresyl fast violet (CFV) dye was changed to 10–50 mg/l. The pH of the investigated solution was altered between 3 and 10 by 0.1 or 0.01 M of Hydrochloric acid and sodium hydroxide. Several variables were investigated, such as pH level, time in contact, initial concentration, agitation rate, dose of adsorbent, and temperature.

The cresyl fast violet (CFV) solution was prepared with a 100 mg/l concentration. This solution was the starting material for preparing other concentrations by diluting it with distilled water. The compound CFV, with a molecular formula of C_18_H_16_ClN_3_O and a molecular weight of 325.792 g/mol, was provided by Dayang Chem (Hangzhou) Co., Ltd. The beakers, which held the specified amount of adsorbent and CFV concentration, were swirled for a predetermined duration and at a specific agitation speed, depending on the studied variable. The adsorbent was isolated from all samples using a 45 μm polyethylene membrane filter, and the analysis was performed thrice. The concentration of CFV remaining in the samples collected at specific time intervals was determined using a UV-Vis spectrophotometer (UVmini-1240, Shimadzu) at a wavelength of 626 nm.

-The following equation was used to determine the percent amount (%) of adsorbate removal:

% Removal =(C_o_-C_e_) /C_o_×100, since C_0_, C_e_ is the concentration of adsorbates at initial and after adsorption in (mg/L), respectively^12^.

-The following equation can be used to get the capacity of adsorption, q_e_ (mg/ g): q_max_ = [(C_0_ –C_e_)V] / m ×(v/w), V(L) is the volume, and m (g) is the weight of the dry adsorbent^12^.

The collected adsorption data at various solution temperatures were utilized to investigate the kinetics and the corresponding thermodynamic parameters. An investigation was carried out to study the adsorption isotherms using various initial MV concentrations as the basis for the analysis.

### Statistical studies

The dye elimination parameters were modeled and optimized by ANOVA as well as design expert programs^67^. These studies depend on an empirical simulation to forecast the outcomes of reactions when substances are removed. The adsorption-based removal approach (RDA), which depends on these programs, was the best possible strategy due to its ability to construct a prototype with minimal required expertise. Furthermore, it reduces both the time and expenses associated with experiments.

## Conclusion

In this work, it is shown that the prepared mullite nanoceramic material is an effective adsorbent for the removal of cresyl fast violet dye from aqueous solutions. Some parameters of initial dye concentration, adsorbent dosage, time, pH, and stirring effect for dye removal were investigated. The collected information assured that mullite nanoceramic material is a suitable adsorbent for CFV dye with a removal efficiency of 99.46% after 30 min at room temperature, 600 rpm, and an initial dye concentration of 10 ppm at pH 7. The Freundlich isotherm model was used to fit the pseudo-second-order model. The thermodynamic analysis revealed that the adsorption of CFV dye onto nano mullite was spontaneous and endothermic, as indicated by the established values of ΔH^o^, ΔS^o^, and ΔG^o^ under the given experimental conditions. The desorption study of CFV dye using HCl or NaOH showed excellent regenerative efficiency. So, these results provide compelling evidence that nano mullite is effective for CFV dye removal with minimal contact time and dosage with high removal and regenerative efficiency.

## Electronic supplementary material

Below is the link to the electronic supplementary material.

Supplementary Material 1

## Data Availability

All data generated or analyzed during this study are included in this published article [and its supplementary information files].
