# Supplementary material for: Kinetics and process optimization studies for the effective removal of cresyl fast violet dye using reusable nanosized mullite
Source: Sci Rep. 2024 Dec 31;14:32164. doi: 10.1038/s41598-024-81653-y (PMC11688456; doi:10.1038/s41598-024-81653-y)
Supplement: Supplementary file 1 — Supplementary Material 1 [file 41598_2024_81653_MOESM1_ESM.docx]

**Kinetics and process optimization studies for the effective removal of Cresyl Fast Violet dye using reusable nanosized mullite**

## Omar A. Fouad ^1*^, Yara M. Adly^1^, Wafaa M. Hosny ^1^, Gehad G. Mohamed^1,2^, Maysa R. Mostafa^1*^

1. Chemistry Department, Faculty of Science, Cairo University, 12613, Giza, Egypt.
2. Nanoscience Department, Basic and Applied Sciences Institute, Egypt-Japan University of Science and Technology, New Borg El Arab, Alexandria, 21934, Egypt

*** Omar A. Fouad: The** **corresponding author**

E-mail: [oahmed@sci.cu.edu.eg](mailto:oahmed@sci.cu.edu.eg)

***Maysa R. Mostafa: The corresponding author**

E-mail: [Maysaramadan140@cu.edu.eg](mailto:Maysaramadan140@cu.edu.eg)

1. **Materials**

**2.1. Materials and solutions**

All items were employed at the analytical-grade level and used precisely as intended. Sigma-Aldrich Chemie GmbH, located in Eschenstrasse 5 D-82024 TAUFKIRCHEN, is the source of the chemicals ethyl alcohol, tetraethyl orthosilicate, and aluminum chloride hexahydrate. The German company Riedel-deHaen supplied the ammonia solution. Sodium hydroxide (NaOH) and hydrochloric acid (HCl) were bought from (Honeywell-Germany). Without any additional purification, the basic dye (Cresyl fast Violet) was purchased from Sigma-Aldrich and utilized as supplied. Cresyl fast Violet (CFV) pigment stock solutions were made by distilled water. By dilution, the desired concentrations were achieved.

1. **Instruments**

With Ni-filtered Cu K radiation (= 1.5406), the phase composition of the generated nanoparticles and sintered samples was determined using a Bruker D8 Discover X-ray diffractometer. In gas adsorption studies, N_2_ has been used as the adsorptive gas to determine the BET surface area at 77 K. Before the adsorption test, the materials were evacuated under a high vacuum for four to twelve hours. The calculation was based on the Brunauer-Emmett-Teller (BET) hypothesis, and the analysis was carried out using a Nova Touch LX2 analyzer. a scanning electron microscope (SEM) of cracked surfaces using the Philips XL30 model, an accelerating voltage of 30 kV, magnification up to 400000, and resolution for W [3.5 nm], the microstructure and pore size distribution of a few selected samples were investigated. Before testing, samples received a thin layer of gold coating. The shape and size of the generated nanoparticles were examined using transmission electron microscopy (TEM; JEOL JEM-2100, Tokyo, Japan). A spectrophotometric approach was used to determine the amounts of the dyes under investigation. Using a UV-vis spectrophotometer, calibration curves for the dye were created by plotting their absorbance against concentration at a maximum wavelength of 626 nm for cresyl fast violet. Using the Beer- Lambert equation, the final dye concentration was calculated spectrophotometrically to correspond to the dye λ_max_[1].

Supplementary Figure 1. N_2_ adsorption–desorption isotherms for nano mullite.


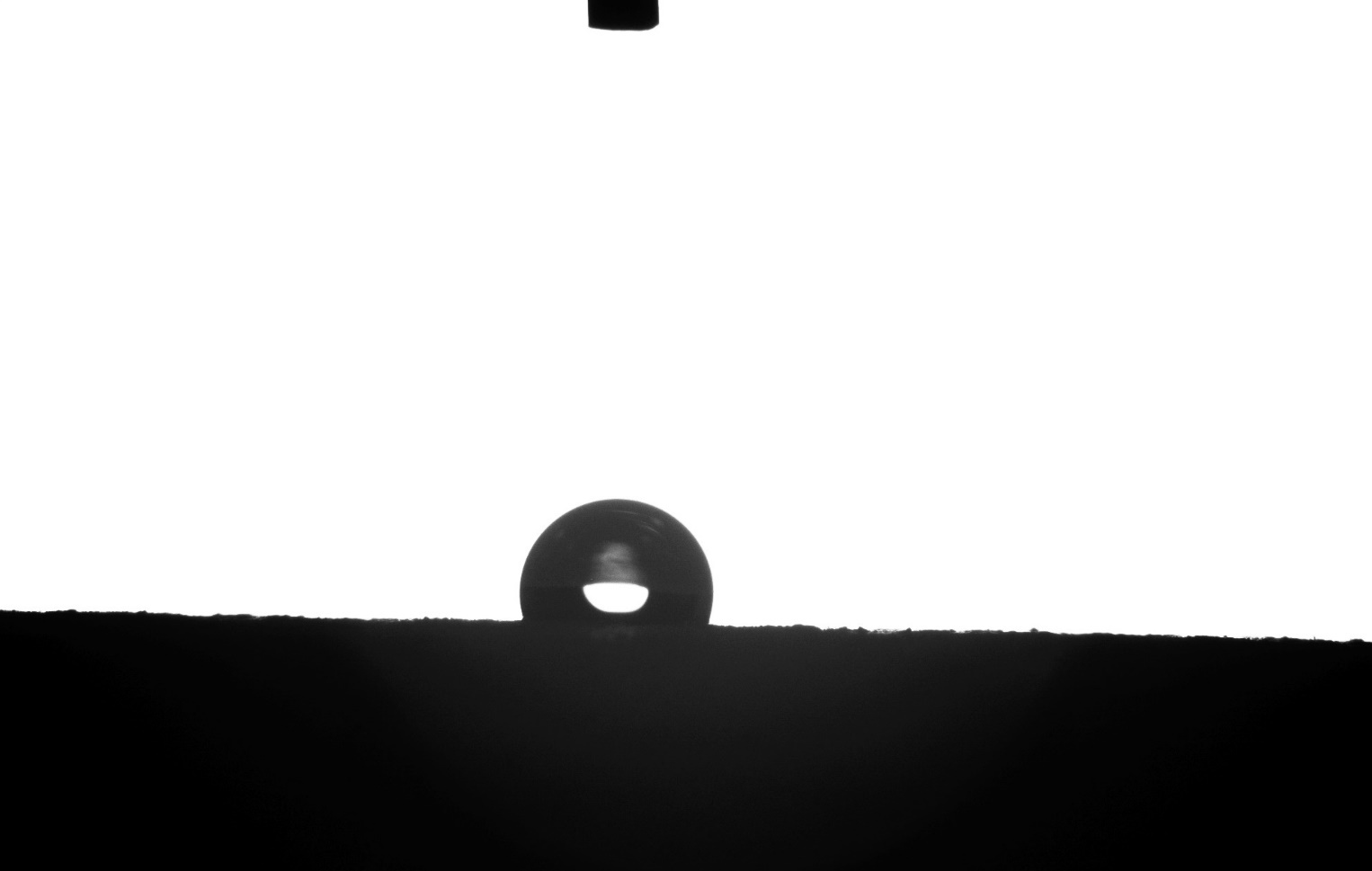


Supplementary Figure 2. contact angle measurement of nano mullite.

***
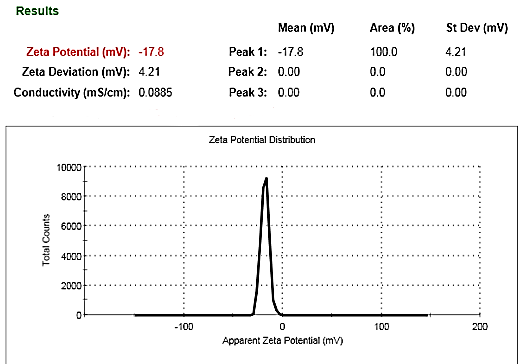
***

Supplementary Figure 3. Zeta potential for nano mullite.

Supplementary Figure 4.The adsorption of CFV on nano mullite using van’t Hoff plot.

Supplementary Figure 5. N_2_ adsorption–desorption isotherms for nano mullite with CFV

.

**References**

1. Kumar, V. and K.D. Gill, *Basic concepts in clinical biochemistry: a practical guide*. 2018: Springer.
